# Heterogeneity of p53 dependent genomic responses following ethanol exposure in a developmental mouse model of fetal alcohol spectrum disorder

**DOI:** 10.1371/journal.pone.0180873

**Published:** 2017-07-19

**Authors:** Maria Camargo Moreno, Sandra M. Mooney, Frank A. Middleton

**Affiliations:** 1 Department of Biochemistry & Molecular Biology, Upstate Medical University, Syracuse, NY, United States of America; 2 Department of Pediatrics, University of Maryland School of Medicine, Baltimore, MD, United States of America; 3 Developmental Exposure Alcohol Research Center, Binghamton University, Binghamton, NY, United States of America; 4 Department of Psychiatry & Behavioral Sciences, Upstate Medical University, Syracuse, NY, United States of America; 5 Department of Neuroscience & Physiology, Upstate Medical University, Syracuse, NY, United States of America; Nathan S Kline Institute, UNITED STATES

## Abstract

Prenatal ethanol exposure can produce structural and functional deficits in the brain and result in Fetal Alcohol Spectrum Disorder (FASD). In rodent models acute exposure to a high concentration of alcohol causes increased apoptosis in the developing brain. A single causal molecular switch that signals for this increase in apoptosis has yet to be identified. The protein p53 has been suggested to play a pivotal role in enabling cells to engage in pro-apoptotic processes, and thus figures prominently as a hub molecule in the intracellular cascade of responses elicited by alcohol exposure. In the present study we examined the effect of ethanol-induced cellular and molecular responses in primary somatosensory cortex (SI) and hippocampus of 7-day-old wild-type (WT) and p53-knockout (KO) mice. We quantified apoptosis by active caspase-3 immunohistochemistry and ApopTag™ labeling, then determined total RNA expression levels in laminae of SI and hippocampal subregions. Immunohistochemical results confirmed increased incidence of apoptotic cells in both regions in WT and KO mice following ethanol exposure. The lack of p53 was not protective in these brain regions. Molecular analyses revealed a heterogeneous response to ethanol exposure that varied depending on the subregion, and which may go undetected using a global approach. Gene network analyses suggest that the presence or absence of p53 alters neuronal function and synaptic modifications following ethanol exposure, in addition to playing a classic role in cell cycle signaling. Thus, p53 may function in a way that underlies the intellectual and behavioral deficits observed in FASD.

## Introduction

Fetal Alcohol Spectrum Disorder (FASD) is caused by maternal alcohol consumption during pregnancy. Abstaining from consumption would prevent this disorder, unfortunately this has proven difficult to achieve. The general U.S. prevalence rate of FASD is on average 3.6% of children [1–2]. The effects of ethanol on the developing brain include a wide range of molecular alterations, including changes in DNA, RNA, and protein. Knowledge of the mechanisms underlying these alterations may help redress the damage. One major consequence of developmental ethanol exposure is a marked increase in apoptosis that strongly contributes to the FASD phenotype (reviewed in [3]. Moreover, there appears to be an enhanced vulnerability to cell death and apoptosis in specific brain regions, including the anterior vermis of the cerebellum, the hippocampus, the corpus callosum, the striatum, and selected regions of the frontal, parietal and temporal lobes [4–7].

At the protein level, several molecules have been identified with putative roles in directing the cell towards apoptosis, including neurotrophins, phosphoinositide 3-kinase, p53, phosphatase and tensin homolog, gamma-Aminobutyric acid and glutamate receptors, and membrane recycling machinery [8–9]. Among these, the transcription factor p53 has emerged from work in our lab as well as others, as being particularly affected by ethanol exposure [10–12]. p53 is a well-studied protein that has been nicknamed the ‘guardian of the genome’ because of its classical function as a transcription factor that induces the transcription of p21 leading to a stoppage of the cell cycle upon detection of cellular stress [13]. Transcribed p21 goes on to inhibit cell cycle progression by binding to Cdk2 [14]. Inhibition of Cdk2 prevents cells in G1 from progressing into the next cell cycle phase which is when the synthesis of new DNA occurs. p53 is also recognized to prevent cell cycle progression in other phases of the cell cycle [15]. In addition to p53’s classic role as cell cycle inhibitor, it is also well accepted that it promotes expression of genes involved in apoptosis, DNA repair, senescence, autophagy, and development [16–17]. The focus of the current study is on the role of p53 in apoptosis signaling.

The protein Bax is one of several well-studied examples of an apoptosis genes that p53 promotes transcription of [18–21]. Studies of the role of p53 in alcohol-induced apoptosis, however, reveal a range of reported outcomes regarding its level of expression. The majority of ethanol studies have focused singularly on the liver, and have in general found that p53 protein and mRNA increases, although there are studies that show the opposite trend [22–27]. Studies of p53 that have considered the damage done to the brain in drinking paradigms are fewer [11–12, 28].

To better understand whether p53 coordinates the observed increase in apoptosis in the brain in response to alcohol exposure, the present study was undertaken to provide a comprehensive analysis of the molecular mechanisms that underlie the contribution of p53 to the FASD phenotype. We focused in particular on two of the brain areas described above: the primary somatosensory cortex and the hippocampus. We took a unique approach in examining total RNA expression levels in primary somatosensory cortex in combination with a more spatially-refined RNA expression analysis related to p53 pathways in individual subregions of the somatosensory cortex (lamina 2–3, 4, 5, 6) and subregions of the hippocampus (CA1 and dentate gyrus).

Our results reveal that there is a heterogeneous response to alcohol exposure that varies depending on the brain subregion under consideration, which may go undetected using a global measurement approach. We conclude that in the brain regions assessed, the absence of p53 does not protect against ethanol-induced apoptosis. Instead we suggest that p53 plays a more specialized role in neuronal function; specifically influencing gene networks related to the development of neuronal projections, in addition to its classic role in cell cycle signaling. Moreover, we also identified changes in gene networks involved in fatty acid and ke tone body metabolism that are influenced by the presence of p53 following ethanol exposure.

## Results

### Assessment of apoptosis in a developmental mouse FASD model

Our use of a developmental ethanol exposure model in P7 mice enabled us to determine if there were significant increases in apoptosis using two different techniques (ApopTag and active caspase 3 immunoreactivity). We found that although there were increases, the results differed in magnitude for each method.

#### ApopTag

**Fig 1:**
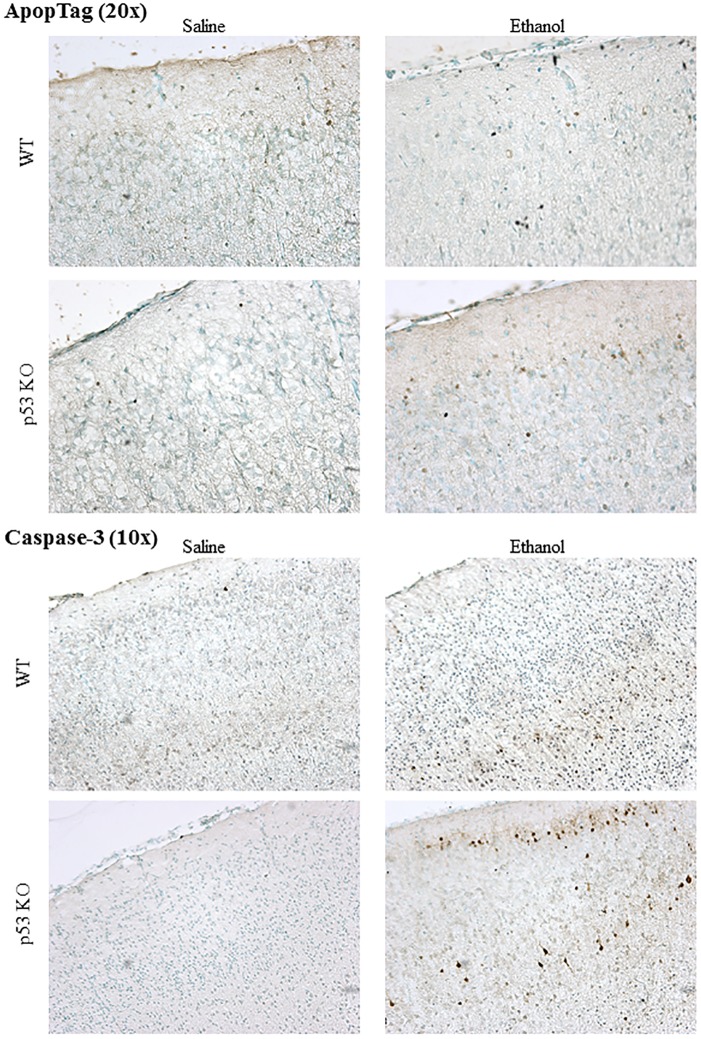
Cell labeled by ApopTag or active caspase-3 immunostaining in somatosensory cortex 8 hours after ethanol exposure in 7-day-old mice. Upper, ApopTag staining (viewed at 20x) appeared as small nuclear puncta easily distinguished from background and counter-staining when present, which tended to be most evident in upper cortical layers. Lower, Immunostaining for active caspase 3 labeled mostly whole cell somas that appeared darkly stained and were most evident in both upper and lower cortical layers in some cases.

**Fig 2:**
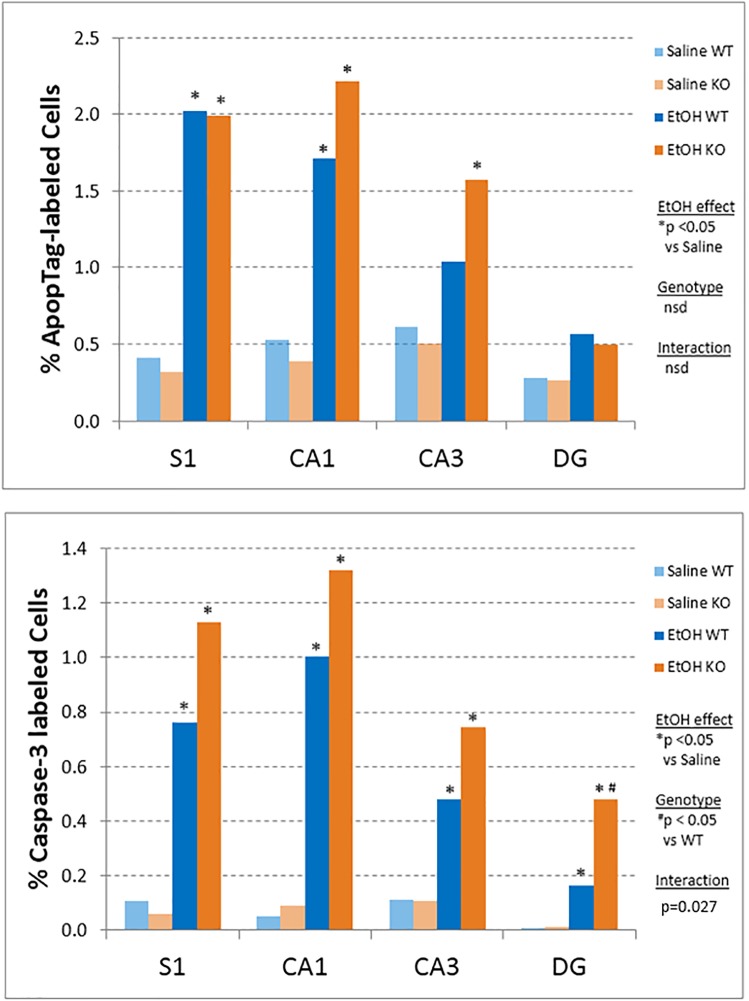
Quantification of apoptosis in whole somatosensory cortex (S1) and three subregions of the hippocampal formation (CA1, CA3, dentate gyrus (DG)) following ethanol administration. Upper, The percentage of ApopTag immunopositive cells was determined. An average of 0.93% of cells were labeled across both treatment groups in the four brain regions. Lower, Counting of active caspase-3 immunopositive cells revealed an average of 0.41% of cells labeled across the treatment groups in the four brain regions of interest, and a significant main effect of alcohol treatment (p < 0.05) in all four brain regions, as well as a significant main effect of Genotype in the DG and a significant Treatment x Genotype interaction in the DG. Asterisks indicate p < 0.05 compared to the Saline-treated mice within each Genotype. Pound (#) symbol indicates significant effect (p < 0.05) of Genotype.

ApopTag staining appeared as small nuclear puncta that was easily distinguished from background and counter-staining when present (Fig 1, top). In some cases, this staining was most evident in upper cortical layers. Overall, an average of 0.93% of cells were labeled in the ApopTag assays across the treatment groups in the four brain regions of interest (S1, CA1, CA3, and DG). Comparison of cell counts in the different groups using two-way ANOVA revealed a significant main effect of alcohol treatment (p < 0.05) in three of the brain regions (S1, CA1, DG), but no effect of Genotype and no Treatment x Genotype interaction in any brain region. Looking specifically at the effects of ethanol in the mice confirmed significant increases in ApopTag staining as a result of ethanol exposure in three brain regions in KO mice (S1, CA1, CA3) and two brain regions in WT mice (S1, CA1) (Fig 2, top; Table 1). Some evidence for increased ApopTag staining was observed in the other areas, even though the difference was not significant (Fig 2, top). There were no between group differences in ApopTag staining due to Genotype in any brain region in these post-hoc comparisons.

**Table 1:** Percentage labeled cells following ethanol or saline injections.

**ApopTag**	**S1**	**CA1**	**CA3**	**DG**
Saline WT	0.41	0.53	0.61	0.28
Saline KO	0.32	0.39	0.51	0.27
EtOH WT	2.02	1.71	1.04	0.57
EtOH KO	1.99	2.21	1.58	0.50
**Active Caspase-3**	**S1**	**CA1**	**CA3**	**DG**
Saline WT	0.11	0.05	0.11	0.01
Saline KO	0.06	0.09	0.11	0.01
EtOH WT	0.76	1.00	0.48	0.17
EtOH KO	1.13	1.32	0.75	0.48
Spearman Correlation ApopTag: Caspase-3	0.679	0.756	0.680	0.516

#### Active caspase 3

Immunostaining for active caspase 3 mostly labeled whole cell somas that appeared very darkly stained, and were most evident in both upper and lower cortical layers in some cases (Fig 1, bottom). Overall, an average of 0.41% of cells were labeled in the active caspase 3 assays across the treatment groups in the four brain regions of interest (S1, CA1, CA3, and DG). Comparison of cell counts in the different groups using two-way ANOVA revealed a significant main effect of alcohol treatment (p < 0.05) in all four brain regions, as well as a significant main effect of Genotype in the DG and a significant Treatment x Genotype interaction in the DG. Between group comparisons confirmed significant increases in staining as a result of ethanol exposure in each of the brain regions regardless of Genotype (Fig 2, bottom; Table 1).

Direct comparison of the ApopTag and active caspase 3 staining in the same animals was performed using a Spearman's correlation test. This indicated the presence of significantly correlated counts with both assay techniques, with the greatest similarity in counts observed for the CA1 region (Table 1).

### Ethanol alters cell cycle, synaptic, and metabolic genes in a p53-related manner in whole somatosensory cortex

After rigorous data cleaning and alignment, the results from the global assessment of RNA expression changes in whole somatosensory cortex yielded an average of 12 M reads aligned to RefSeq. Most (87%) of these reads were fully contained within an exon, 0.94% were partly within an exon, 6.26% were intronic, and 5.71% were intergenic. The RNA-Seq data were analyzed using a two-way ANOVA with a focus on the two main factors of Exposure (ethanol vs saline) and p53 Genotype (KO vs WT) as well as the interaction between them (Exposure x Genotype).The results indicated that 824 genes showed nominally significant main effects of Genotype (uncorrected P < 0.05), 763 genes had nominally significant main effects of Treatment (uncorrected P-value < 0.05), and 1969 genes had nominally significant interactions of Genotype x Treatment (uncorrected P-value < 0.05).

To set the stage for understanding which genes of those affected by ethanol might be differentially affected in WT and KO mice, we first briefly describe the genes that were most significantly affected by ethanol in each individual post-hoc test analyzed by genotype. In the WT mice, the genes most significantly affected by ethanol included Salt inducible kinase 2 (Sik2), Cyclin D binding myb-like transcription factor 1 (Dmtf1), and Microtubule-associated protein 4 (Map4) (Table 2).

**Table 2:** Genes with significant changes in expression after ethanol treatment in P7 WT mouse somatosensory cortex.

							*2-way ANOVA*			*Post-Hoc Contrast*				
							*Treatment*, *Genotype*, *Interaction*			*WT* ^*ETOH*^ *vs*. *WT* ^*Saline*^				
										Mean Expression				* *
	Gene ID	Gene Name	Chr	Start	Stop	Strand	p-value (Treatment)	p-value (Genotype)	p-value (Genotype x Treatment)	WT ^ETOH^	WT ^Saline^	Fold Chg	p-value	FDR
1	Sik2	salt inducible kinase 2	9	50892801	51009074	-	0.00019	0.00065	0.00002	4.06	1.28	3.17	0.00000	0.07
2	Dmtf1	cyclin D binding myb-like transcription factor 1	5	9118868	9161777	-	0.00023	0.00044	0.00013	12.57	3.35	3.76	0.00001	0.08
3	Map4	microtubule-associated protein 4	9	109931774	110083955	+	0.00415	0.00005	0.00002	7.82	1.79	4.36	0.00002	0.08
4	Cit	citron	5	115845656	116006342	+	0.00044	0.00037	0.00025	2.39	0.23	10.64	0.00003	0.08
5	Smarca2	SWI/SNF related, matrix associated, actin depend-ent regulator of chromatin, subfamily a, member 2	19	26605160	26778322	+	0.00157	0.00103	0.00009	11.85	1.40	8.46	0.00003	0.08
6	Prune2	prune homolog 2 (Drosophila)	19	16956118	17223933	+	0.00128	0.64599	0.00016	3.32	1.48	2.25	0.00004	0.08
7	Hp1bp3	heterochromatin protein 1, binding protein 3	4	138216612	138244683	+	0.00069	0.07633	0.00035	19.89	10.49	1.90	0.00004	0.08
8	Il17d	interleukin 17D	14	57524829	57543167	+	0.00104	0.56984	0.00025	3.91	10.66	-2.73	0.00005	0.08
9	Rnf6	ring finger protein (C3H2C3 type) 6	5	146209194	146221458	-	0.00091	0.06118	0.00035	4.37	0.15	28.45	0.00005	0.08
10	Rasgrf1	RAS protein-specific guanine nucleotide-releasing factor 1	9	89909775	90026980	+	0.00009	0.08841	0.00645	12.45	1.04	11.95	0.00006	0.08
11	Fam13b	family with sequence similarity 13, member B	18	34442351	34506824	-	0.00673	0.00144	0.00011	18.43	4.63	3.98	0.00007	0.08
12	Dnajc18	DnaJ (Hsp40) homolog, subfamily C, member 18	18	35671105	35703145	-	0.00179	0.01027	0.00031	12.57	7.85	1.60	0.00007	0.08
13	Grik2	glutamate receptor, ionotropic, kainate 2 (beta 2)	10	49099463	49788755	-	0.00042	0.35360	0.00133	11.77	3.68	3.20	0.00007	0.08
14	Ncoa7	nuclear receptor coactivator 7	10	30645582	30803108	-	0.00653	0.01225	0.00018	8.78	2.27	3.87	0.00009	0.10
15	Plcb4	phospholipase C, beta 4	2	135741830	136013069	+	0.00241	0.06248	0.00065	6.71	0.58	11.53	0.00013	0.12
16	Golga1	golgi autoantigen, golgin subfamily a, 1	2	39016156	39065542	-	0.00351	0.00162	0.00053	3.95	0.58	6.84	0.00013	0.12
17	Sirt3	sirtuin 3 (silent mating type information regulation 2, homolog) 3 (S. cerevisiae)	7	140863663	140881870	-	0.00534	0.95473	0.00045	1.82	12.18	-6.69	0.00015	0.12
18	Ctu1	cytosolic thiouridylase subunit 1 homolog (S. pombe)	7	43672031	43678298	+	0.00024	0.04411	0.01268	2.69	5.58	-2.07	0.00015	0.12
19	Sh3gl2	SH3-domain GRB2-like 2	4	85205456	85389380	+	0.06213	0.00381	0.00009	101.15	60.55	1.67	0.00016	0.12
20	Syne1	synaptic nuclear envelope 1	10	5020196	5194708	-	0.00235	0.00243	0.00117	4.55	0.49	9.31	0.00017	0.13
21	Nudt8	nudix (nucleoside diphosphate linked moiety X)-type motif 8	19	4000580	4002103	+	0.08048	0.21373	0.00009	6.16	14.22	-2.31	0.00019	0.13
22	Sorbs2	sorbin and SH3 domain containing 2	8	45507788	45827907	+	0.00437	0.29684	0.00074	3.22	0.42	7.67	0.00019	0.13
23	Hivep3	human immunodeficiency virus type I enhancer binding protein 3	4	119814678	120135412	+	0.03469	0.00036	0.00019	1.55	0.27	5.78	0.00021	0.13
24	Txlng	taxilin gamma	X	162778917	162829455	-	0.01230	0.00360	0.00041	1.94	0.25	7.76	0.00022	0.13
25	Nop9	nucleolar protein homolog (Yeast), pumilio domain-containing Protein	14	55745693	55755635	+	0.00282	0.00809	0.00155	3.80	9.19	-2.42	0.00023	0.13

Results sorted by post-hoc significance in the WT samples. The # genes with P values ≤ 0.05 was 2,038 out of 15,248 queried. N_total_ = 24 mice.

Notably, compared to the p53WT mice, different genes showed nominally significant effects of ethanol in the KO mice (Table 3). In this case, the four most affected genes included Jun dimerization protein 2 (Jdp2), ATPase, aminophospholipid transporter (APLT), class I, type 8A, member 1 (Atp8a1), and Eukaryotic translation initiation factor 4E member 2 (Eif4E2) (Table 3).

**Table 3:** Genes with significant expression changes after ethanol treatment in P7 KO mouse somatosensory cortex.

							*2-way ANOVA*			*Post-Hoc Contrast*				
							*Treatment*, *Genotype*, *Interaction*			*KO* ^*ETOH*^ *vs*. *KO* ^*Saline*^				
										Mean Expression				
** **	Gene ID	Gene Name	Chr	Start	Stop	Strand	p-value (Treatment)	p-value (Genotype)	p-value (Genotype x Treatment)	KO ^ETOH^	KO ^Saline^	Fold change	P-value	FDR
1	Jdp2	Jun dimerization protein 2	12	85599105	85639879	+	0.00474	0.82156	0.00826	4.64	2.10	2.21	0.00082	0.93
2	Atp8a1	ATPase, aminophospholipid transporter (APLT), class I, type 8A, member 1	5	67618139	67847432	-	0.03356	0.05795	0.00150	3.48	6.90	-1.98	0.00088	0.93
3	Eif4e2	eukaryotic translation initiation factor 4E member 2	1	87213914	87228859	+	0.05460	0.00681	0.00190	5.65	0.26	21.68	0.00135	0.93
4	Nars	asparaginyl-tRNA synthetase	18	64499647	64516558	-	0.00021	0.18884	0.75149	19.79	34.18	-1.73	0.00145	0.93
5	Sqle	squalene epoxidase	15	59315092	59331194	+	0.00050	0.11345	0.37065	44.86	64.81	-1.44	0.00167	0.93
6	Aloxe3	arachidonate lipoxygenase 3	11	69126377	69149116	+	0.00158	0.38422	0.15555	3.65	2.46	1.49	0.00223	0.93
7	Trub2	TruB pseudouridine (psi) synthase homolog 2 (E. coli)	2	29774684	29787672	-	0.01909	0.02822	0.01274	0.46	1.53	-3.35	0.00252	0.93
8	Stx16	syntaxin 16	2	174077051	174099772	+	0.00424	0.95722	0.07782	2.47	1.12	2.22	0.00291	0.93
9	Plekhb1	pleckstrin homology domain containing, family B (evectins) member 1	7	100643896	100658457	-	0.00077	0.02222	0.52186	1.14	3.72	-3.26	0.00303	0.93
10	Stx16	syntaxin 16	2	174077051	174099772	+	0.00403	0.00678	0.09452	1.75	0.99	1.77	0.00317	0.93
11	Ap1g1	adaptor protein complex AP-1, gamma 1 subunit	8	109778583	109864210	+	0.00174	0.01786	0.24411	4.28	7.68	-1.79	0.00322	0.93
12	Necab1	N-terminal EF-hand calcium binding protein 1	4	14952245	15149132	-	0.07970	0.70533	0.00491	40.03	80.49	-2.01	0.00326	0.93
13	Clcn4	chloride channel 4–2	7	7282309	7298837	-	0.00860	0.22439	0.04679	28.77	49.45	-1.72	0.00341	0.93
14	Mob3a	MOB kinase activator 3A	10	80685253	80701821	-	0.01468	0.80982	0.03750	5.26	9.74	-1.85	0.00422	0.93
15	Syt1	synaptotagmin I	10	108497650	109010976	-	0.84295	0.00173	0.00043	41.34	60.54	-1.46	0.00442	0.93
16	Asph	aspartate-beta-hydroxylase	4	9628646	9669163	-	0.00064	0.01087	0.90692	15.71	22.32	-1.42	0.00451	0.93
17	Pih1d1	PIH1 domain containing 1	7	45155946	45160065	+	0.53032	0.00680	0.00135	16.08	7.07	2.27	0.00480	0.93
18	Stard4	StAR-related lipid transfer (START) domain containing 4	18	33201421	33213817	-	0.06998	0.29159	0.00981	2.24	6.27	-2.80	0.00481	0.93
19	Mdk	Midkine	2	91929805	91931703	-	0.22526	0.09691	0.00324	22.18	8.03	2.76	0.00482	0.93
20	Hmgcr	3-hydroxy-3-methylglutaryl-Coenzyme A reductase	13	96648962	96670937	-	0.29823	0.64500	0.00251	32.52	69.15	-2.13	0.00488	0.93
21	Prkar2b	protein kinase, cAMP dependent regulatory, type II beta	12	31958479	32061280	-	0.02067	0.14821	0.03323	53.89	95.40	-1.77	0.00489	0.93
22	Ankrd39	ankyrin repeat domain 39	1	36538171	36547253	-	0.12787	0.02430	0.00591	2.26	0.34	6.68	0.00503	0.93
23	Hsd17b7	hydroxysteroid (17-beta) dehydrogenase 7	1	169949537	169969206	-	0.01304	0.06298	0.05826	2.39	4.93	-2.06	0.00518	0.93
24	Gtf2a1	general transcription factor II A, 1	12	91555262	91590488	-	0.00619	0.00143	0.14021	1.39	3.13	-2.26	0.00553	0.93
25	Jmjd7	Pla2g4b - phospholipase A2, group IVB (cytosolic)	2	120027483	120032605	+	0.81699	0.00388	0.00055	3.62	2.08	1.74	0.00565	0.93

Results sorted according to post-hoc significance in the KO samples. The # genes with P values ≤ 0.05 was 615 out of 15,248 queried.

Because of the apparent differences in the genes altered by ethanol in the two genotypes, we also examined the baseline differences due to genotype in the saline-treated condition of the experimental model. A total of 2,088 genes were significantly changed (Table 4). Interestingly, there was considerable overlap in the genes showing a baseline difference in the two genotypes with those that showed ethanol-induced effects in each genotype separately. In fact, the top three affected genes in the WT mice treated with ethanol vs saline (Table 2) and in the p53 KO vs p53 WT mice (Table 4) overlapped completely (i.e., Map4, Sik2, and Dmtf1). The presence of these overlapping genes was reinforced by the results of the two-way ANOVA interaction analysis, which specifically detects genes whose expression changes due to ethanol are strongly influenced by p53 genotype (and vice versa). As expected, the Map4, Sik2, and Dmtf1 genes all showed significant interaction effects (Table 5). Thus, many genes altered due to ethanol in WT mice are also altered due to baseline p53 genotype differences. Most of these genes do not belong to canonical p53 signaling pathways, although several are clearly involved in p53-related processes, including chromatin and cell cycle regulation (e.g., Dmtf1, Hivep3, and Smarca2) as well as synaptic function (e.g., Sh3gl2).

**Table 4:** Genes with significant expression changes due to genotype in saline-treated P7 WT and KO mice.

							*2-way ANOVA*			*Post-Hoc Contrast*				
							*Treatment*, *Genotype*, *Interaction*			*KO* ^*Saline*^ *vs*. *WT* ^*Saline*^				
										Mean Expression				
	Gene ID	Gene Name	Chr	Start	Stop	Strand	p-value (Treatment)	p-value (Genotype)	p-value (Genotype x Treatment)	KO ^Saline^	WT ^Saline^	Fold change	P-value	FDR
1	Map4	microtubule-associated protein 4	9	109931774	110083955	+	0.00415	0.00005	0.00002	12.45	1.79	6.94	0.00000	0.03
2	Sik2	salt inducible kinase 2	9	50892801	51009074	-	0.00019	0.00065	0.00002	3.75	1.28	2.93	0.00001	0.06
3	Dmtf1	cyclin D binding myb-like transcription factor 1	5	9118868	9161777	-	0.00023	0.00044	0.00013	11.88	3.35	3.55	0.00002	0.06
4	Hivep3	human immunodeficiency virus type I enhancer binding protein 3	4	119814678	120135412	+	0.03469	0.00036	0.00019	3.09	0.27	11.51	0.00002	0.06
5	Smarca2	SWI/SNF related, matrix associated, actin dependent regulator of chromatin, subfamily a, member 2	19	26605160	26778322	+	0.00157	0.00103	0.00009	12.62	1.40	9.00	0.00002	0.06
6	Cit	citron	5	115845656	116006342	+	0.00044	0.00037	0.00025	2.40	0.23	10.66	0.00003	0.06
7	Axl	AXL receptor tyrosine kinase	7	25756500	25788734	-	0.19396	0.00014	0.00092	0.86	5.69	-6.61	0.00003	0.06
8	Fam13b	family with sequence similarity 13, member B	18	34442351	34506824	-	0.00673	0.00144	0.00011	21.27	4.63	4.59	0.00003	0.06
9	Sh3gl2	SH3-domain GRB2-like 2	4	85205456	85389380	+	0.06213	0.00381	0.00009	110.79	60.55	1.83	0.00004	0.07
10	Anks1b	ankyrin repeat and sterile alpha motif domain containing 1B	10	90575727	90972985	+	0.00120	0.00009	0.00672	3.50	0.83	4.22	0.00006	0.09
11	Mrpl55	mitochondrial ribosomal protein L55	11	59202486	59206136	+	0.17417	0.00025	0.00255	3.60	12.35	-3.43	0.00007	0.10
12	Pcbp2	poly(rC) binding protein 2	15	102470632	102500060	+	0.02222	0.00011	0.00870	7.31	17.81	-2.44	0.00008	0.10
13	Syt1	synaptotagmin I	10	108497650	109010976	-	0.84295	0.00173	0.00043	60.54	29.84	2.03	0.00008	0.10
14	Golga1	golgi autoantigen, golgin subfamily a, 1	2	39016156	39065542	-	0.00351	0.00162	0.00053	4.57	0.58	7.91	0.00009	0.10
15	Txlng	taxilin gamma	X	162778917	162829455	-	0.01230	0.00360	0.00041	2.40	0.25	9.62	0.00012	0.10
16	Tppp	tubulin polymerization promoting protein	13	74009419	74035754	+	0.17454	0.00235	0.00065	28.08	9.17	3.06	0.00012	0.10
17	Ncoa7	nuclear receptor coactivator 7	10	30645582	30803108	-	0.00653	0.01225	0.00018	8.49	2.27	3.75	0.00012	0.10
18	Syt1	synaptotagmin I	10	108497650	109010976	-	0.47102	0.00270	0.00060	131.82	61.28	2.15	0.00013	0.10
19	Dync1li2	dynein, cytoplasmic 1 light intermediate chain 2	8	104417674	104443048	-	0.00764	0.00232	0.00070	32.04	9.70	3.30	0.00013	0.10
20	Jmjd7	Pla2g4b - phospholipase A2, group IVB (cytosolic)	2	120027483	120032605	+	0.81699	0.00388	0.00055	2.08	5.78	-2.78	0.00014	0.11
21	Alad	aminolevulinate, delta-dehydratase	4	62505984	62519910	-	0.01717	0.00408	0.00056	1.65	5.50	-3.33	0.00015	0.11
22	Dnajc18	DnaJ (Hsp40) homolog, subfamily C, member 18	18	35671105	35703145	-	0.00179	0.01027	0.00031	11.86	7.85	1.51	0.00016	0.11
23	Syne1	synaptic nuclear envelope 1	10	5020196	5194708	-	0.00235	0.00243	0.00117	3.73	0.49	7.65	0.00018	0.12
24	Camk1d	calcium/calmodulin-dependent protein kinase ID	2	5293457	5676047	-	0.08877	0.00444	0.00074	15.46	6.34	2.44	0.00019	0.12
25	Shank2	SH3/ankyrin domain gene 2	7	144175520	144422676	+	0.02071	0.00228	0.00145	7.46	1.02	7.29	0.00019	0.12

Results sorted according to post-hoc significance in the saline-treated KO vs WT samples. The # genes with P values ≤ 0.05 was 2,088.

**Table 5:** Genes with significant interaction effects in expression after ethanol treatment in P7 WT and KO mice.

							*2-way ANOVA Interaction*		*Post-Hoc Contrasts of Interest*					
							*Genotype x Treatment*		*WT* ^*EtOH*^ *vs WT* ^*Saline*^		*KO* ^*EtOH*^ *vs KO* ^*Saline*^		*KO* ^*Saline*^ *vs WT* ^*Saline*^	
	Gene ID	Gene Name	Chr	Start	Stop	Strand	P-value	FDR	Fold Chg	P-value	Fold Chg	P-value	Fold Chg	P-value
1	Sik2	salt inducible kinase 2	9	50892801	51009074	-	0.00002	0.15	3.17	0.00000	-1.21	0.11781	2.93	0.00001
2	Map4	microtubule-associated protein 4	9	109931774	110083955	+	0.00002	0.15	4.36	0.00002	-1.77	0.00777	6.94	0.00000
3	Sh3gl2	SH3-domain GRB2-like 2	4	85205456	85389380	+	0.00009	0.27	1.67	0.00016	-1.31	0.00681	1.83	0.00004
4	Smarca2	SWI/SNF related, matrix associated, actin dependent regulator of chromatin, subfamily a, member 2	19	26605160	26778322	+	0.00009	0.27	8.46	0.00003	-1.63	0.10869	9.00	0.00002
5	Nudt8	nudix (nucleoside diphosphate linked moiety X)-type motif 8	19	4000580	4002103	+	0.00009	0.27	-2.31	0.00019	1.63	0.00616	-2.23	0.00030
6	Fam13b	family with sequence similarity 13, member B	18	34442351	34506824	-	0.00011	0.27	3.98	0.00007	-1.51	0.04162	4.59	0.00003
7	Dmtf1	cyclin D binding myb-like transcription factor 1	5	9118868	9161777	-	0.00013	0.29	3.76	0.00001	-1.07	0.71778	3.55	0.00002
8	Prune2	prune homolog 2 (Drosophila)	19	16956118	17223933	+	0.00016	0.29	2.25	0.00004	-1.13	0.23131	1.54	0.00234
9	Ncoa7	nuclear receptor coactivator 7	10	30645582	30803108	-	0.00018	0.29	3.87	0.00009	-1.48	0.07524	3.75	0.00012
10	Hivep3	human immunodeficiency virus type I enhancer binding protein 3	4	119814678	120135412	+	0.00019	0.29	5.78	0.00021	-2.15	0.02382	11.51	0.00002
11	Cit	citron	5	115845656	116006342	+	0.00025	0.30	10.64	0.00003	-1.14	0.72748	10.66	0.00003
12	Il17d	interleukin 17D	14	57524829	57543167	+	0.00025	0.30	-2.73	0.00005	1.10	0.41739	-1.66	0.00407
13	Mvb12a	Fam125a - family with sequence similarity 125, member A	8	71542930	71548027	+	0.00027	0.30	-2.11	0.00061	1.52	0.01173	-2.06	0.00061
14	Dnajc18	DnaJ (Hsp40) homolog, subfamily C, member 18	18	35671105	35703145	-	0.00031	0.30	1.60	0.00007	-1.07	0.33251	1.51	0.00016
15	Hp1bp3	heterochromatin protein 1, binding protein 3	4	138216612	138244683	+	0.00035	0.30	1.90	0.00004	-1.04	0.69045	1.58	0.00049
16	Rnf6	ring finger protein (C3H2C3 type) 6	5	146209194	146221458	-	0.00035	0.30	28.45	0.00005	-1.34	0.58522	12.50	0.00044
17	Txlng	taxilin gamma	X	162778917	162829455	-	0.00041	0.30	7.76	0.00022	-1.71	0.10678	9.62	0.00012
18	Nme4	NME/NM23 nucleoside diphosphate kinase 4	17	26091745	26095471	-	0.00041	0.30	-2.91	0.00069	1.79	0.02198	-3.08	0.00048
19	Syt1	synaptotagmin I	10	108497650	109010976	-	0.00043	0.30	1.51	0.00296	-1.46	0.00442	2.03	0.00008
20	Macrod2	MACRO domain containing 2	2	140395430	142390051	+	0.00043	0.30	4.03	0.00041	-1.82	0.04721	4.36	0.00031
21	Sirt3	sirtuin 3 (silent mating type information regulation 2, homolog) 3 (S. cerevisiae)	7	140863663	140881870	-	0.00045	0.30	-6.69	0.00015	1.53	0.20943	-3.16	0.00351
22	Zfp410	zinc finger protein 410	12	84316859	84343831	+	0.00045	0.30	-7.90	0.00048	3.04	0.04164	-4.14	0.00466
23	Celf1	CUGBP, Elav-like family member 1	2	90940397	91019498	+	0.00045	0.30	-2.31	0.00039	1.39	0.05613	-1.91	0.00193

Results sorted according to significance of the interaction effect in the 2-way ANOVA model. The # genes with P values ≤ 0.05 was 2,144.

#### Gene ontology enrichment analysis

To formally examine the numerous genes with robust interaction effects, we performed two levels of biological pathway analysis. The first was a widely-used enrichment approach based on over-representation in specific KEGG Gene Ontology pathways. The results from this analysis indicate enrichment of changed genes in pathways related to metabolic, synaptic, and cell cycle functions, including: Butanoate metabolism (KEGG ID 157, p = 0.00017); GABAergic synapse (KEGG ID 90, p = 0.00229), and Chronic myeloid leukemia (KEGG ID 215, p = 0.00298). Notably, each of the nominally enriched pathways contained between 4–20% of the genes mapping to that pathway (Table 6).

**Table 6:** KEGG pathways enriched with genes showing significant Ethanol x Genotype interaction effects.

		Enrichment		KEGG pathway Genes			Score per 2-way ANOVA factors			Score per Post-Hoc Contrasts		
	KEGG Pathway Name	Score	p-value	*# genes in list*, *in pathway*	*# genes not in list*, *in pathway*	*% genes in pathway that are present*	*Treatment*	*Genotype*	*Treatment x Genotype*	*WT*^*ETOH*^ *vs*. *WT*^*SALINE*^	*KO*^*ETOH*^ *vs*. *KO*^*SALINE*^	*KO*^*SALINE*^ *vs*. *WT*^*SALINE*^
1	Butanoate metabolism (KEGG ID:157)	8.71	0.00017	5	22	18.52	1.39	0.45	2.55	2.64	0.91	1.78
2	GABAergic synapse (KEGG ID:90)	6.08	0.00229	7	85	7.61	1.63	0.40	2.40	2.86	0.46	1.30
3	Chronic myeloid leukemia (KEGG ID:215)	5.82	0.00298	6	66	8.33	0.81	0.79	2.51	2.40	1.04	2.26
4	Estrogen signaling pathway (KEGG ID:13)	5.78	0.00309	7	90	7.22	0.99	0.71	2.45	2.47	0.89	2.06
5	Osteoclast differentiation (KEGG ID:24)	5.65	0.00353	8	118	6.35	1.39	0.58	2.40	2.71	0.75	1.77
6	T cell receptor signaling pathway (KEGG ID:132)	5.39	0.00455	7	97	6.73	0.79	0.48	2.37	2.29	0.93	2.00
7	Proteoglycans in cancer (KEGG ID:260)	5.31	0.00495	11	213	4.91	0.93	0.46	2.38	2.39	0.85	1.96
8	Phosphatidylinositol signaling system (KEGG ID:211)	5.17	0.00567	6	76	7.32	1.12	0.33	2.31	2.44	0.69	1.58
9	Protein processing in endoplasmic reticulum (KEGG ID:35)	5.16	0.00573	9	157	5.42	0.73	0.68	2.33	2.19	0.99	2.07
10	Ras signaling pathway (KEGG ID:19)	5.15	0.00583	11	218	4.80	0.85	0.79	2.41	2.22	1.08	2.18
11	Oocyte meiosis (KEGG ID:7)	5.14	0.00589	7	102	6.42	0.77	0.42	2.21	2.00	1.01	1.80
12	ErbB signaling pathway (KEGG ID:212)	4.83	0.00797	6	82	6.82	0.82	0.82	2.50	2.40	1.02	2.29
13	Insulin signaling pathway (KEGG ID:64)	4.81	0.00817	8	137	5.52	0.75	0.68	2.44	2.32	1.01	2.13
14	Terpenoid backbone biosynthesis (KEGG ID:258)	4.71	0.00901	3	19	13.64	0.44	0.16	2.53	1.32	2.20	1.86
15	Dorso-ventral axis formation (KEGG ID:207)	4.71	0.00901	3	19	13.64	0.80	0.88	2.23	1.79	1.24	2.26
16	Fc epsilon RI signaling pathway (KEGG ID:121)	4.50	0.01112	5	63	7.35	0.90	0.34	2.50	2.45	0.96	1.93
17	Taurine and hypotaurine metabolism (KEGG ID:217)	4.14	0.01594	2	8	20.00	2.01	0.23	2.35	3.07	0.23	1.35
18	Valine, leucine and isoleucine degradation (KEGG ID:40)	4.00	0.01825	4	47	7.84	0.87	0.76	2.51	2.24	1.22	2.12
19	N-Glycan biosynthesis (KEGG ID:30)	4.00	0.01825	4	47	7.84	0.26	0.20	2.50	1.95	1.51	1.87
20	FoxO signaling pathway (KEGG ID:195)	3.99	0.01855	7	129	5.15	0.73	0.56	2.54	2.31	1.17	2.18
21	Endometrial cancer (KEGG ID:272)	3.94	0.01947	4	48	7.69	1.02	0.42	2.52	2.54	0.88	2.00
22	beta-Alanine metabolism (KEGG ID:18)	3.86	0.02117	3	27	10.00	2.17	0.34	2.45	3.22	0.18	1.33
23	TNF signaling pathway (KEGG ID:111)	3.81	0.02212	6	104	5.45	1.17	0.33	2.37	2.54	0.68	1.85
24	Non-small cell lung cancer (KEGG ID:273)	3.75	0.02344	4	51	7.27	1.02	0.42	2.52	2.54	0.88	2.00
25	Epstein-Barr virus infection (KEGG ID:149)	3.73	0.02398	9	201	4.29	1.28	0.88	2.32	2.59	0.58	2.22
26	Toxoplasmosis (KEGG ID:213)	3.69	0.02488	6	107	5.31	1.19	0.43	2.49	2.65	0.77	2.05
27	Circadian rhythm (KEGG ID:146)	3.68	0.02513	3	29	9.38	0.31	0.28	2.23	1.85	1.18	1.81
28	Progesterone-mediated oocyte maturation (KEGG ID:168)	3.62	0.02681	5	80	5.88	1.20	0.38	2.28	2.43	0.67	1.65
29	Alanine, aspartate, glutamate metabolism (KEGG ID:190)	3.60	0.02724	3	30	9.09	2.17	0.34	2.45	3.22	0.18	1.33
30	Adrenergic signaling in cardiomyocytes (KEGG ID:259)	3.51	0.02978	7	143	4.67	1.21	0.35	2.40	2.47	0.80	1.81
31	Neurotrophin signaling pathway (KEGG ID:128)	3.37	0.03448	6	116	4.92	0.72	0.85	2.50	2.27	1.14	2.31
32	mTOR signaling pathway (KEGG ID:216)	3.37	0.03449	4	58	6.45	0.95	0.42	2.43	2.35	0.95	1.93
33	Inositol phosphate metabolism (KEGG ID:219)	3.37	0.03449	4	58	6.45	1.06	0.26	2.15	2.29	0.63	1.68
34	Chemokine signaling pathway (KEGG ID:158)	3.36	0.03457	8	181	4.23	1.06	0.77	2.56	2.59	0.93	1.99
35	Morphine addiction (KEGG ID:160)	3.28	0.03751	5	88	5.38	1.26	0.41	2.31	2.59	0.60	1.25
36	Glioma (KEGG ID:122)	3.22	0.04002	4	61	6.15	0.95	0.98	2.61	2.55	1.03	2.42
37	Renal cell carcinoma (KEGG ID:16)	3.17	0.04196	4	62	6.06	1.08	0.73	2.45	2.22	1.11	2.17
38	RNA transport (KEGG ID:105)	3.16	0.04248	7	155	4.32	0.81	0.91	2.43	2.20	1.09	2.34
39	Vascular smooth muscle contraction (KEGG ID:233)	3.14	0.04341	6	123	4.65	1.05	0.88	2.34	2.44	0.76	1.61
40	RIG-I-like receptor signaling pathway (KEGG ID:106)	3.12	0.04396	4	63	5.97	0.50	0.45	2.24	2.01	1.04	1.70
41	Aldosterone-regulated sodium reabsorption (KEGG ID:72)	3.11	0.04465	3	37	7.50	1.23	0.21	2.55	2.70	0.76	1.82
42	Hepatitis C (KEGG ID:108)	3.01	0.04911	6	127	4.51	1.10	0.70	2.52	2.61	0.85	2.25

Results sorted according to significance of the enrichment score. A total of 7,387 KEGG pathways were queried, with 42 showing p < 0.05.

#### Pathway ANOVA analysis

**Fig 3:**
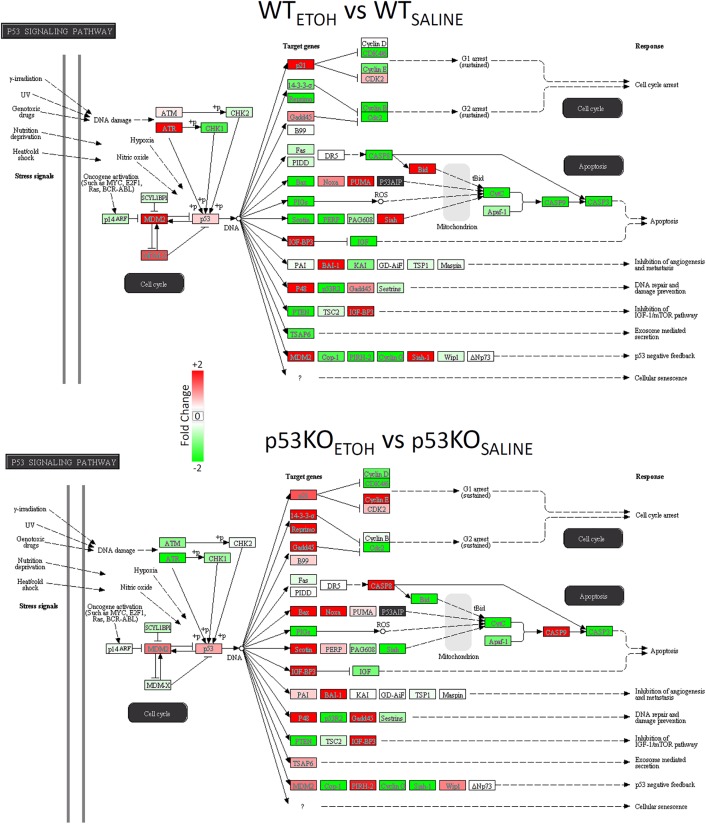
Changes in p53 Signaling genes due to ethanol. Ethanol induced changes in all n = 117 transcripts in the KEGG p53 Signaling pathway are shown. Colors denote direction of change (red = increased, green = decreased, white = no change).

We followed up the enrichment analysis with a Pathway ANOVA analysis in Partek Genomics Suite using the KEGG annotation database. The results indicated a significant overall main effect of ethanol treatment on the net expression of six KEGG transcript groups (Steroid biosynthesis; p53 signaling pathway; Collecting duct acid secretion; Glycosphingolipid biosynthesis; Valine, leucine and isoleucine biosynthesis; and mTOR signaling; Table 7). Among these, we note that the p53 signaling pathway ranked 2^nd^ according to significance (p = 0.0262), contained 117 transcripts, and exhibited significant decreases in WT animals exposed to ethanol (-1.24 fold, p = 0.0177) and a trend for increased expression in KO mice versus WT mice in the saline condition (2.21 fold, p = 0.0837); Fig 3, upper). Another pathway of interest (Apoptosis, ranked 17^th^) did not show a significant overall effect, but did show notable decreases in the KO mice exposed to ethanol (-1.22 fold, p = 0.0278; Table 7).

**Table 7:** Top Pathway ANOVA KEGG gene sets showing effects due to main effect of ethanol vs saline treatment.

			*2-way ANOVA*			*Post-Hoc Contrasts*					
			*Treatment*, *Genotype*, *Interaction*			*WT*^*EtOH*^ *vs WT*^*Saline*^		*KO*^*EtOH*^ *vs KO*^*Saline*^		*KO*^*Saline*^ *vs WT*^*Saline*^	
	Top Shifted KEGG Pathways	# Genes	p value (Genotype)	p value (Treatment)	p value (Genotype x Treatment)	p value	Fold Change	p value	Fold Change	p value	Fold Change
1	**Steroid biosynthesis**	23	0.2038	**0.0158**	0.7359	0.0924	-1.20	**0.0429**	-1.29	0.4853	1.23
2	**p53 signaling pathway**	117	0.2273	**0.0262**	0.1758	**0.0177**	-1.24	0.4073	-1.07	0.0837	2.21
3	**Collecting duct acid secretion**	34	0.1023	**0.0267**	**0.0115**	**0.0029**	1.28	0.7056	-1.02	**0.0069**	1.88
4	**Glycosphingolipid biosynthesis—lacto and neolacto series**	51	0.1555	**0.0330**	0.1318	**0.0169**	-1.36	0.5446	-1.07	0.0508	1.49
5	**Valine, leucine and isoleucine biosynthesis**	5	0.9468	**0.0472**	0.6489	0.2226	-1.23	0.0816	-1.37	0.7116	-1.67
6	**mTOR signaling pathway**	128	0.3904	**0.0492**	0.3224	0.3986	-1.05	**0.0443**	-1.14	0.9199	2.76
7	Hematopoietic cell lineage	155	0.5511	0.0529	0.5811	0.2651	-1.43	0.0791	-1.71	0.4218	11.85
8	Ubiquitin mediated proteolysis	252	0.0896	0.0666	0.7680	0.2348	-1.03	0.1244	-1.05	0.2836	2.60
9	Terpenoid backbone biosynthesis	30	0.8003	0.0761	**0.0124**	0.4301	1.06	**0.0059**	-1.27	**0.0396**	1.64
10	Thyroid cancer	63	0.8149	0.0898	**0.0226**	**0.0100**	1.43	0.5475	-1.07	0.1060	3.05
11	Small cell lung cancer	161	0.4736	0.1002	0.2305	0.7025	-1.02	0.0562	-1.11	0.7096	2.73
12	Sphingolipid metabolism	79	0.4761	0.1008	0.3915	0.0868	-1.30	0.5213	-1.10	0.2760	1.40
13	Ubiquinone and other terpenoid-quinone biosynthesis	11	0.0540	0.1058	**0.0154**	**0.0086**	-1.39	0.4043	1.10	**0.0055**	-1.38
14	Pantothenate and CoA biosynthesis	25	0.5423	0.1125	0.6701	0.1543	-1.15	0.3709	-1.09	0.4676	1.34
15	Aminoacyl-tRNA biosynthesis	67	0.6971	0.1187	0.3145	0.0812	-1.18	0.6462	-1.04	0.6485	1.24
16	Rheumatoid arthritis	117	0.3522	0.1406	0.1875	0.0613	1.19	0.8943	1.01	0.1242	3.13
17	Apoptosis	156	0.2257	0.1412	0.0627	0.7184	1.03	**0.0278**	-1.22	0.5653	2.34
18	Type I diabetes mellitus	70	0.5160	0.1434	0.0856	**0.0351**	3.39	0.8170	-1.09	0.0989	18.38
19	beta-Alanine metabolism	38	0.3524	0.1488	0.2449	0.0784	1.19	0.8146	1.02	0.8546	1.47
20	Retinol metabolism	104	0.1425	0.1632	0.1413	0.0554	-1.53	0.9474	1.02	0.0502	2.86
21	Pentose and glucuronate interconversions	41	0.1020	0.1745	0.1989	0.0752	-1.37	0.9509	-1.01	0.0508	1.17
22	Degradation of aromatic compounds	4	0.7231	0.1800	0.7221	0.2300	-1.23	0.4588	-1.14	0.6171	1.41
23	Selenocompound metabolism	31	0.2463	0.1974	0.4634	0.1625	-1.16	0.6650	-1.05	0.1909	1.10
24	Synthesis and degradation of ketone bodies	13	0.1026	0.1995	**0.0016**	**0.0486**	1.32	**0.0026**	-1.58	**0.0017**	2.55
25	Caffeine metabolism	7	0.1695	0.2067	0.3732	0.1399	-2.81	0.7684	-1.60	0.1210	-2.61

Results sorted according to significance of the ethanol treatment p-value. Of the 255 KEGG pathways were queried, 6 had p < 0.05.

**Fig 4:**
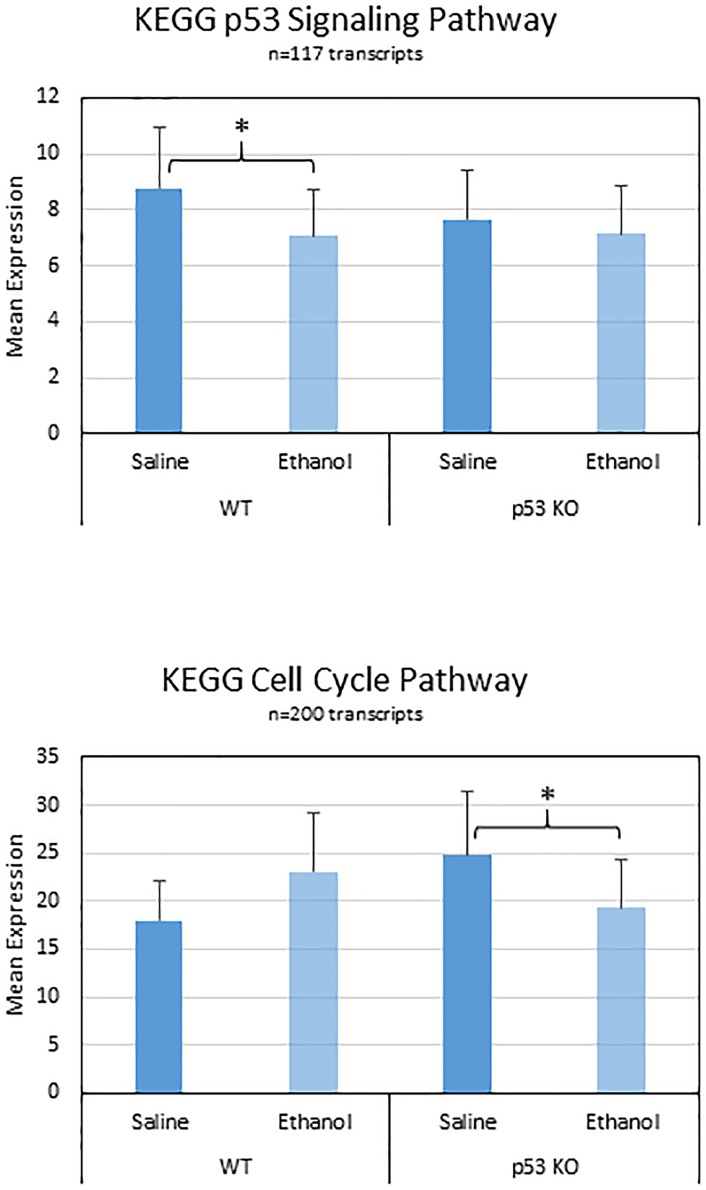
Ethanol-induced changes in the p53 Signaling Pathway and Cell Cycling Pathway. The transcripts contained with each of these KEGG pathways were averaged for each condition. There were trends for decreased expression in the p53 Signaling in the WT mice (upper) and for differential responses to ethanol in the WT and p53 KO mice (lower). Cell Cycle changes were confirmed by a significant Genotype x Treatment interaction from the Pathway ANOVA results. Bars show mean, T-bars depict standard error of the mean. Asterisks indicate significant (p<0.05) post-hoc contrasts within each genotype for the ethanol versus saline comparison.

We next examined the Pathway ANOVA results to elucidate the most robust baseline differences between p53 KO and WT mice in the saline treatment condition. We identified a single KEGG pathway with a significant overall effect of Genotype on expression (SNARE interactions in vesicular transport), along with several neurotransmission-related KEGG pathways that all showed evidence of increased transcript expression in p53 KO vs WT mice (Table 8). The pathways with significant post-hoc changes included SNARE interactions in vesicular transport (+2.88-fold), Neurotrophin signaling (+10.68-fold), Axon guidance (+8.09-fold), and Nicotine addiction (+2.46-fold). The individual gene changes in p53-related transcripts following ethanol exposure in the two different genotypes was made evident after visualization of the expression data in a heat map within the KEGG p53 signaling pathway itself (Fig 3). This indicated that there were more transcripts with decreased expression in the WT mice following ethanol treatment (Fig 4, top). We also observed several individual transcripts with opposite patterns of change due to ethanol in the two genotypes, including Bax, Casp8, and Casp9 (decreased in WT, increased in KO), as well as ATR and ATM (increased in WT, decreased in KO).

**Table 8:** Top Pathway ANOVA KEGG gene sets showing effects due to the main effect of genotype.

			*2-way ANOVA*			*Post-Hoc Contrasts*					
			*Treatment*, *Genotype*, *Interaction*			*WT*^*EtOH*^ *vs WT*^*Saline*^		*KO*^*EtOH*^ *vs KO*^*Saline*^		*KO*^*Saline*^ *vs WT*^*Saline*^	
	Top SHIFTED KEGG Pathways	# Genes	p value (Genotype)	p value (Treatment)	p value (Genotype x Treatment)	p value	Fold Change	p value	Fold Change	p value	Fold Change
1	SNARE interactions in vesicular transport	58	**0.0131**	0.8376	0.0553	0.1211	1.14	0.1892	-1.09	**0.0050**	2.88
2	Ubiquinone and other terpenoid-quinone biosynthesis	11	0.0540	0.1058	**0.0154**	0.0086	-1.39	0.4043	1.10	**0.0055**	-1.38
3	Histidine metabolism	41	0.0698	0.4350	0.3924	0.2571	-1.11	0.9550	1.01	0.0670	1.33
4	Ubiquitin mediated proteolysis	252	0.0896	0.0666	0.7680	0.2348	-1.03	0.1244	-1.05	0.2836	2.60
5	Lipoic acid metabolism	4	0.0921	0.2374	0.3080	0.1329	-1.43	0.8973	-1.04	0.0666	-1.76
6	Salivary secretion	146	0.0971	0.5055	**0.0071**	0.0164	1.50	0.0754	-1.26	**0.0048**	8.61
7	Synaptic vesicle cycle	110	0.1019	0.3117	0.2946	0.1582	1.20	0.9772	-1.00	0.0690	1.92
8	Pentose and glucuronate interconversions	41	0.1020	0.1745	0.1989	0.0752	-1.37	0.9509	-1.01	0.0508	1.17
9	Collecting duct acid secretion	34	0.1023	0.0267	**0.0115**	0.0029	1.28	0.7056	-1.02	**0.0069**	1.88
10	Allograft rejection	64	0.1024	0.6223	0.0806	0.1136	-1.51	0.3237	1.41	**0.0263**	1.77
11	Synthesis and degradation of ketone bodies	13	0.1026	0.1995	**0.0016**	0.0486	1.32	**0.0026**	-1.58	**0.0017**	2.55
12	Neurotrophin signaling pathway	253	0.1057	0.8440	**0.0123**	0.0420	1.37	0.0658	-1.27	**0.0074**	10.68
13	Axon guidance	251	0.1145	0.7451	**0.0359**	0.1614	1.18	0.0782	-1.21	**0.0162**	8.09
14	Nicotine addiction	66	0.1235	0.9641	0.0291	0.1025	1.19	0.0927	-1.18	**0.0148**	2.46
15	ErbB signaling pathway	166	0.1238	0.7213	0.0921	0.1451	1.16	0.3068	-1.09	**0.0332**	2.51
16	Pertussis	115	0.1247	0.9263	**0.0084**	0.0439	1.45	**0.0356**	-1.39	**0.0063**	9.79
17	Autoimmune thyroid disease	80	0.1251	0.6858	0.0865	0.1319	-1.49	0.3097	1.43	**0.0320**	2.00
18	Folate biosynthesis	17	0.1251	0.2229	0.1314	0.0666	-1.49	0.8062	1.06	**0.0432**	1.00
19	Lysine degradation	89	0.1271	0.6357	0.6306	0.5028	1.04	0.9959	-1.00	0.1580	2.13
20	Retinol metabolism	104	0.1425	0.1632	0.1413	0.0554	-1.53	0.9474	1.02	0.0502	2.86
21	Amyotrophic lateral sclerosis (ALS)	85	0.1433	0.8988	0.1794	0.2900	1.04	0.3711	-1.04	0.9172	2.12
22	Graft-versus-host disease	66	0.1435	0.7292	0.0967	0.1519	-1.47	0.3130	1.43	**0.0383**	1.91
23	Intestinal immune network for IgA production	62	0.1438	0.7593	0.1235	0.1875	-1.37	0.3498	1.34	**0.0458**	1.53
24	Ribosome	162	0.1507	0.4106	0.1416	0.1152	-1.24	0.6044	1.08	0.0523	-1.01
25	Phosphatidylinositol signaling system	142	0.1522	0.9724	**0.0095**	0.0417	1.55	**0.0451**	-1.42	**0.0079**	13.54

Results sorted by significance of the genotype p-value. Of the 255 KEGG pathways were queried, only 1 had p < 0.05 for the overall effect.

**Fig 5:**
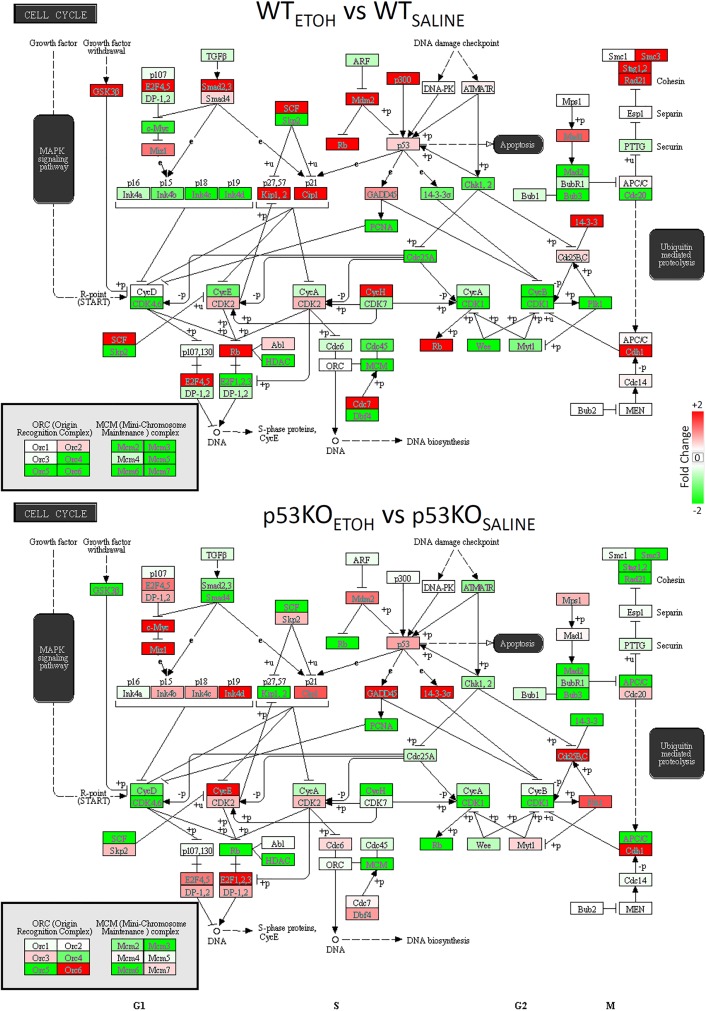
Changes in Cell Cycle genes due to ethanol. Overall, ethanol exposure altered more genes in this KEGG pathway in WT mice than p53 KO mice. Several of the genes that were increased in WT mice after ethanol were decreased in p53 KO, and vice versa. Same convention as Fig 3.

Finally, we examined the Pathway ANOVA results for evidence of significant Ethanol Treatment x Genotype interaction effects. This indicated nominally significant interaction effects in a total of 50 KEGG pathways (Table 9). The two most robustly affected pathways included Butanoate metabolism and Synthesis and degradation of ketone bodies, which were both increased WT mice exposed to ethanol and decreased in KO mice exposed to ethanol. Other KEGG pathways of interest due to their involvement in brain function or p53 signaling that displayed significant interaction effects included: Amphetamine addiction, Long-term potentiation, Neurotrophin signaling, Cell cycle (Fig 4, bottom), Hedgehog signaling, Dopaminergic synapse, Nicotine addiction, GABAergic synapse, D-Glutamine and D-glutamate metabolism, TGF-beta signaling, and Axon guidance (Table 9). The pattern of individual gene changes in Cell Cycle-related transcripts following ethanol treatment in the two different genotypes became evident after visualization of the expression data in a heat map (Fig 5). We found that WT mice exposed to ethanol showed more decreases in Cell Cycle related transcripts compared to KO mice. Moreover, several of the genes that were increased in the WT mice following ethanol exposure were decreased in the KO mice, and vice versa. Together, these observations indicate that p53 is normally involved in the dynamic responses to ethanol.

**Table 9:** Top Pathway ANOVA KEGG gene sets showing significant Ethanol x Genotype interaction effects.

			*2-way ANOVA*			*Post-Hoc Contrasts*					
			*Treatment*, *Genotype*, *Interaction*			*WT*^*EtOH*^ *vs WT*^*Saline*^		*KO*^*EtOH*^ *vs KO*^*Saline*^		*KO*^*Saline*^ *vs WT*^*Saline*^	
	Top SHIFTED KEGG Pathways	# Genes	p value (Genotype)	p value (Treatment)	p value (Genotype x Treatment)	p value	Fold Change	p value	Fold Change	p value	Fold Change
1	Butanoate metabolism (ID:157)	36	0.1962	0.6126	0.00074	0.0098	1.38	0.0034	-1.43	0.0015	3.55
2	Synthesis and degradation of ketone bodies (ID:63)	13	0.1026	0.1995	0.00157	0.0486	1.32	0.0026	-1.58	0.0017	2.55
3	Progesterone-mediated oocyte maturation (ID:168)	148	0.3624	0.4765	0.00365	0.0094	1.47	0.0474	-1.28	0.0075	6.02
4	Estrogen signaling pathway (ID:13)	175	0.2079	0.8344	0.00475	0.0202	1.54	0.0324	-1.40	0.0060	8.95
5	Oocyte meiosis (ID:7)	182	0.1975	0.9672	0.00559	0.0277	1.43	0.0304	-1.36	0.0065	7.97
6	Vascular smooth muscle contraction (ID:233)	233	0.2515	0.9832	0.00571	0.0302	1.36	0.0288	-1.32	0.0079	10.47
7	Hepatitis B (ID:101)	271	0.4805	0.2697	0.00619	0.1154	1.13	0.0088	-1.25	0.0141	4.13
8	Melanogenesis (ID:91)	182	0.2386	0.7206	0.00651	0.0218	1.55	0.0491	-1.37	0.0083	11.21
9	Salivary secretion (ID:67)	146	0.0971	0.5055	0.00713	0.0164	1.50	0.0754	-1.26	0.0048	8.61
10	GnRH signaling pathway (ID:243)	186	0.2038	0.8910	0.00813	0.0330	1.47	0.0451	-1.36	0.0087	10.81
11	Pertussis (ID:252)	115	0.1247	0.9263	0.00839	0.0439	1.45	0.0356	-1.39	0.0063	9.79
12	Amphetamine addiction (ID:97)	145	0.2389	0.7661	0.00939	0.0306	1.50	0.0604	-1.35	0.0108	9.60
13	Phosphatidylinositol signaling system (ID:211)	142	0.1522	0.9724	0.00951	0.0417	1.55	0.0451	-1.42	0.0079	13.54
14	Insulin signaling pathway (ID:64)	259	0.2884	0.8576	0.00985	0.0547	1.35	0.0363	-1.35	0.0129	11.57
15	Phototransduction (ID:44)	38	0.1949	0.9828	0.01011	0.0466	1.58	0.0443	-1.47	0.0098	7.52
16	Prostate cancer (ID:159)	155	0.4791	0.3898	0.01014	0.0168	1.65	0.1232	-1.30	0.0202	7.12
17	Circadian entrainment (ID:41)	199	0.2619	0.8712	0.01044	0.0387	1.42	0.0560	-1.33	0.0125	9.49
18	Long-term potentiation (ID:174)	119	0.1856	0.9682	0.01082	0.0457	1.45	0.0500	-1.37	0.0099	9.02
19	Collecting duct acid secretion (ID:109)	34	0.1023	0.0267	0.01146	0.0029	1.28	0.7056	-1.02	0.0069	1.88
20	Glioma (ID:122)	110	0.1584	0.9329	0.01147	0.0453	1.51	0.0549	-1.39	0.0093	10.53
21	Tuberculosis (ID:268)	291	0.1736	0.9829	0.01184	0.0498	1.32	0.0523	-1.27	0.0101	11.19
22	Neurotrophin signaling pathway (ID:128)	253	0.1057	0.8440	0.01233	0.0420	1.37	0.0658	-1.27	0.0074	10.68
23	Terpenoid backbone biosynthesis (ID:258)	30	0.8003	0.0761	0.01239	0.4301	1.06	0.0059	-1.27	0.0396	1.64
24	Cell cycle (ID:3)	200	0.3997	0.9180	0.01254	0.0599	1.28	0.0474	-1.28	0.0201	5.24
25	Valine, leucine and isoleucine degradation (ID:40)	63	0.6122	0.5121	0.01295	0.1155	1.18	0.0256	-1.28	0.0305	3.03
26	NOD-like receptor signaling pathway (ID:152)	103	0.2516	0.4220	0.01310	0.0217	1.59	0.1383	-1.26	0.0143	8.06
27	Ubiquinone and other terpenoid-quinone biosynthesis (ID:81)	11	0.0540	0.1058	0.01539	0.0086	-1.39	0.4043	1.10	0.0055	-1.38
28	Hedgehog signaling pathway (ID:55)	77	0.3147	0.3255	0.01587	0.0200	1.39	0.1949	-1.19	0.2000	2.62
29	Dopaminergic synapse (ID:222)	279	0.3451	0.8748	0.01604	0.0533	1.35	0.0762	-1.28	0.0212	8.53
30	Wnt signaling pathway (ID:172)	272	0.8859	0.9960	0.01719	0.0674	1.20	0.0666	-1.20	0.0569	3.83
31	Gastric acid secretion (ID:164)	142	0.2324	0.6897	0.01800	0.0439	1.52	0.1089	-1.31	0.0168	8.86
32	Cytosolic DNA-sensing pathway (ID:134)	93	0.5845	0.8687	0.01860	0.0588	-1.33	0.0855	1.27	0.1317	1.95
33	Circadian rhythm (ID:146)	60	0.6935	0.3892	0.02142	0.2071	1.17	0.0288	-1.37	0.1223	5.00
34	Endometrial cancer (ID:272)	94	0.9056	0.4219	0.02143	0.0309	1.31	0.1940	-1.15	0.0686	2.61
35	Colorectal cancer (ID:278)	121	0.7217	0.7752	0.02234	0.0584	1.21	0.1115	-1.16	0.0539	2.78
36	Mucin type O-Glycan biosynthesis (ID:21)	34	0.4786	0.7100	0.02252	0.1236	1.17	0.0532	-1.24	0.1803	1.57
37	Thyroid cancer (ID:54)	63	0.8149	0.0898	0.02257	0.0100	1.43	0.5475	-1.07	0.1060	3.05
38	Nicotine addiction (ID:42)	66	0.1235	0.9641	0.02911	0.1025	1.19	0.0927	-1.18	0.0148	2.46
39	GABAergic synapse (ID:90)	165	0.7915	0.9546	0.02979	0.0929	1.13	0.1056	-1.13	0.1330	2.22
40	D-Glutamine and D-glutamate metabolism (ID:231)	6	0.7994	0.4689	0.03018	0.0434	1.38	0.2227	-1.19	0.0750	2.00
41	TGF-beta signaling pathway (ID:71)	125	0.9325	0.6480	0.03211	0.1730	1.17	0.0621	-1.26	0.0950	4.41
42	Axon guidance (ID:154)	251	0.1145	0.7451	0.03587	0.1614	1.18	0.0782	-1.21	0.0162	8.09
43	Thiamine metabolism (ID:255)	6	0.2729	0.4636	0.03733	0.0499	-1.29	0.2577	1.16	0.0318	-1.44
44	T cell receptor signaling pathway (ID:132)	212	0.9175	0.2847	0.03897	0.3783	1.07	0.0339	-1.21	0.1339	3.64
45	Hippo signaling pathway (ID:183)	269	0.6327	0.7546	0.04052	0.0864	1.40	0.1727	-1.28	0.0714	5.94
46	Other glycan degradation (ID:9)	29	0.6687	0.9396	0.04232	0.1162	-1.30	0.1374	1.27	0.2014	1.28
47	Bacterial invasion of epithelial cells (ID:175)	148	0.7963	0.7084	0.04667	0.0889	1.25	0.2027	-1.17	0.1014	4.61
48	Linoleic acid metabolism (ID:73)	69	0.8134	0.9527	0.04703	0.1450	-1.38	0.1272	1.42	0.1045	2.12
49	Tight junction (ID:239)	241	0.8380	0.8406	0.04749	0.1094	1.26	0.1702	-1.21	0.1090	5.38
50	Sulfur relay system (ID:162)	15	0.7482	0.5964	0.04964	0.0776	-1.37	0.2487	1.22	0.0986	-1.09

Results sorted according to significance of the interaction p-value. A total of 255 KEGG pathways were queried, with 50 showing p < 0.05.

### Whole somatosensory cortex expression changes cluster in genomic regions

After completing the pathway and functional enrichment analysis, we sought to test if there were specific chromosomal regions targeted by ethanol, regardless of their function, where greater numbers of significantly changed genes were located than expected by chance. This analysis focused on the WT mice and identified 6 genomic regions (p<0.05) that appeared disproportionately targeted (Table 10).

**Table 10:** Genomic regions with enriched ethanol effects in WT mice.

Chr	Start	End	Length	Average P-value	Genes in chromosome region
13	52517606	52882394	364788	0.012	Diras2, Gm2848, Syk, BB123696, Auh
6	148355126	148801076	445950	0.019	Tmtc1, Rps4l, Gm6313 4930528G23Rik, Ipo8
15	33685879	34112552	426673	0.028	Tspyl5, Mtdh
14	26944770	27320961	376191	0.032	Appl1, Hesx1, Il17rd, Arhgef3
12	34450193	35097333	647140	0.041	Hdac9, Prps1l1, Snx13
5	62684312	63729823	1045511	0.042	Arap2, Dthd1, Nwd2

### Increased levels of incomplete p53 transcripts in p53 KO mice

**Fig 6:**
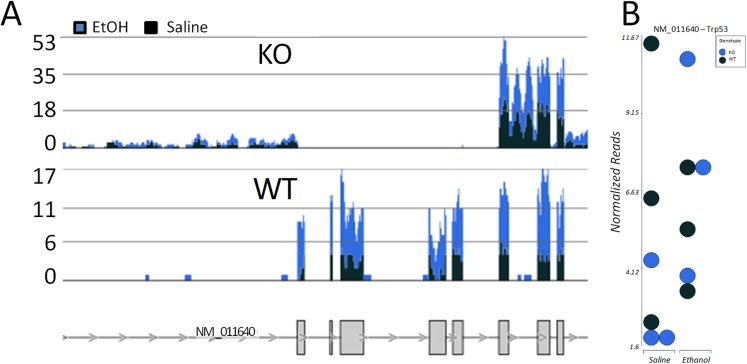
Visualization of p53 expression from the whole somatosensory cortex RNA-Seq data. A, Alignment of RNA-Seq reads spanning the p53 gene, with exon structure shown in gray below. At this magnification, only 8 of the exons can be distinguished (some boxes contain > 1). Note the absence of aligned reads in the p53 KO mice near the 5’ half of the gene, but normal or increased levels of RNA-Seq alignments in the 3’ half of the gene. B, plots of the mean normalized expression level across the entire p53 gene locus for the WT and p53 KO mouse. After ethanol exposure, the levels of the residual p53 gene show evidence of an attempt to increase expression.

While examining the expression data of p53-related genes, we were struck by the observation that the p53 transcript itself was reported as increased in expression in the KO mice. To explore this in more detail, we examined the alignment of p53 transcript reads along the exons of p53 in Partek Flow software. This indicated that while there were higher levels of overall p53 transcripts in the KO mice, the transcripts were not derived from the region that had been deleted by gene- targeted recombination in the creation of the KO mouse line (Fig 6). Instead, there was a notable increase in the aligned read coverage of the last few exons (9–11) of the 3’region of the p53 mRNA in the KO mouse.

### Targeted RNA-sequencing (TREx) confirms findings in somatosensory cortex and reveals heterogeneity in ethanol responses in different cortical and hippocampal subregions

The pathway analysis of the whole somatosensory cortex indicated a close association of p53-related genes with genes involved in the control of cell cycle and neuronal function. These findings reinforce the view of p53 as a central hub in the ethanol-induced dysregulation of brain cellular functions that ultimately leads to either an adaptive survival response or apoptosis. However, the immunohistochemical findings suggested that apoptosis did not occur equally in all cortical layers at the experimental timepoint examined. To examine this further, and provide validation of the initial RNA-Seq results, we performed Targeted RNA-Sequencing (TREx) using a custom panel of 280 p53-related genes involved in neuronal activity, apoptosis, DNA damage and repair, cell cycle regulation, and cell fate, plus housekeeping genes (gene list provided as S1 Table). Specifically, we measured gene expression in RNA samples obtained from somatosensory cortex layers 2–3 (combined), layer 4, layer 5, and layer 6 as well as the dentate gyrus and CA1 regions of the hippocampus.

#### Overall effects

TREx data were analyzed using a two-way ANOVA within each of the subregions to identify genes with significant expression changes due to ethanol Treatment in either WT or KO mice, as well as genes with significant Treatment x Genotype interaction effects. The overall results were grouped into five functional categories based on the TREx design and are summarized in Table 11. Notably, the highest percentage of genes with detectable effects due to Treatment or Treatment x Genotype interactions were found in the p53 Signaling Pathway (11/36 genes changed; 31%), Apoptosis (25/124; 20%), Regulation of Developmental Process (22/99; 22%), and several smaller categories related to neuronal function, including Synaptic Transmission (6/14; 43%) and Regulation of Long-Term Synaptic Plasticity (3/5; 60%).

**Table 11:** Combined summary of TREx expression changes due to ethanol in different brain regions, by function.

GENE ONTOLOGY CATEGORY	# Genes in Ontology	# Genes selected for TREx	Δ Genes	Genes from combined areas that changed in custom TRex assay (p < 0.05)
KEGG 04115: p53 signaling pathway	69	36	11	ATM, BAI1, CCNE2, CDK1, CDK4, EI24, PTEN, RPRM, SESN1, SESN2, TSC2
GO:0006915 Apoptosis	1698	124	25	ATM, BCL2, BCL2L1, BIRC5, CASP2, CASP6, CASP8, CD27, CDK1, CDKN1B, CRADD, CTGF, DLX1, EI24, FOXO3, GAD1, JUN, KRAS, NR2E1, PAK7, PTEN, SOX9, TRP53BP, XPA, YWHAE
GO:0007049 Cell Cycle	1421	133	17	ATM, BCL2, BIRC5, CDK1, CDKN1B, CTGF, DDB1, JUN, LIG3, MAPK1, PPP3CA, PTEN, RPRM, SESN1, SMC1A, TRP53BP2, TSC2
GO:0006974 Response to DNA damage stimulus	641	119	13	APEX2, ATM, BCL2, CDK1, DDB1, FOXO3, LIG3, MAPK1, PARP1, SMC1A, TDG, XPA, XRCC4
GO:0006281 DNA repair	402	87	9	APEX2, ATM, DDB1, LIG3, PARP1, SMC1A, TDG, XPA, XRCC4
GO:0050767 Regulation of neurogenesis	787	43	8	BCL2, DLX1, DLX2, HES1, MIB1, NR2E1, PTEN, XRCC4
GO:0030182 Neuron differentiation	1308	54	8	BCL2, DLX1, DLX2, HES1, MIB1, NR2E1, PTEN, SMAD4
GO:0007268 Synaptic transmission	562	14	6	EGR1, GAD1, KRAS, PPP3CA, PTEN, SYP
GO:0043005 Neuron projection	1169	44	6	CASP8, GAD1, MAPK1, PTEN, TSC2, YWHAE
GO:0048169 Regulation of long-term neuronal synaptic plasticity	33	5	3	EGR1, KRAS, SYP
GO:0045321 Leukocyte activation	768	38	6	ATM, BCL2, CD27, EGR1, FOXP1, XRCC4
GO:0050793 Regulation of developmental process	2369	99	22	BAI1, BCL2, CAMK1, CASP6, CD27, CDK1, CDKN1B, DLX1, DLX2, ERRFI1, FOXO3, HES1, JUN, MAPK1, MIB1, NR2E1, PTEN, SMAD1, SMAD4, SOX9, STAT1, XRCC4
GO:0016477 Cell migration	1177	35	7	BCL2, CTGF, GAD1, NR2E1, PTEN, SST, YWHAE

Colors indicate functional classes: Green, p53-related genes; Red, apoptosis-related genes; Yellow, cell cycle-related genes; Orange, DNA damage and repair; Blue, Neuronal and immune

#### Heterogeneity of ethanol-induced effects

**Fig 7:**
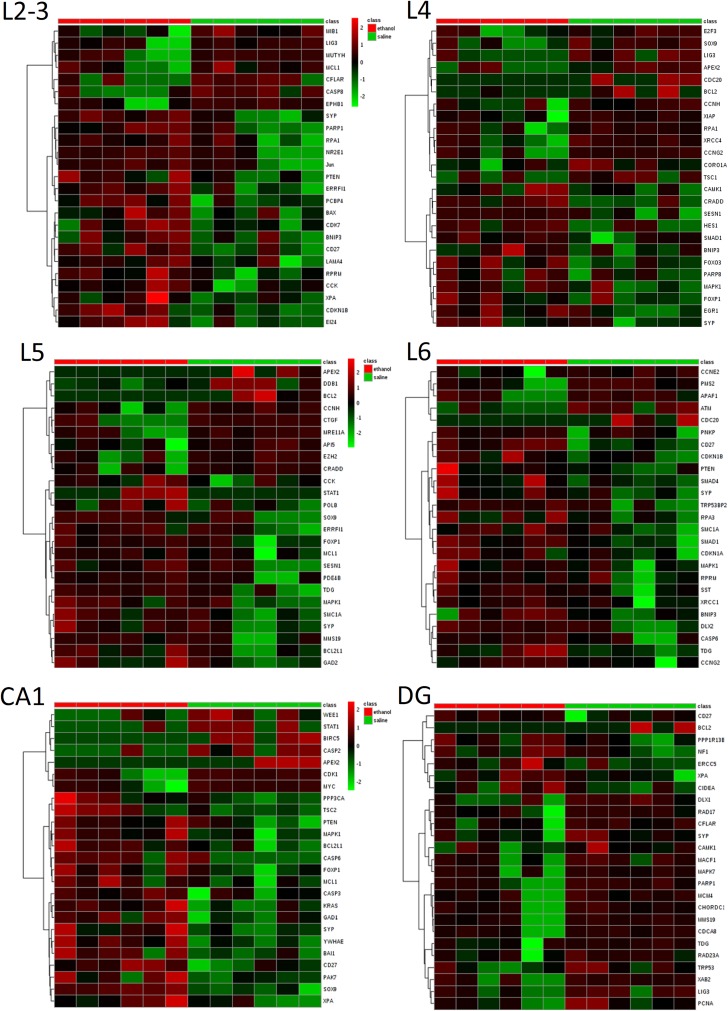
Heterogeneous responses of p53-related genes in different brain regions after ethanol exposure in WT mice. Hierarchical cluster maps show the 25 most significantly changed genes in each area ranked by t test. Each column represents the data from one mouse brain region sample (n = 6 per treatment group). Note that the pattern of change in each subregion distinguishes the ethanol and saline treated samples, but largely involves different genes.

Our overall results indicated that ethanol exposure triggers gene expression changes in the five functional categories of interest related to p53 and neuronal signaling (Table 11). However, we also noted that the specific genes with expression changes were not the same in each subregion in the WT and KO mice exposed to ethanol. For example, nine genes in the p53 Signaling Pathway were changed in WT mice exposed to ethanol, but the specific genes with changes were unique for each area, with Layer 5 and the dentate gyrus containing no significantly changed genes, Layer 4 and Layer 6 containing one changed gene, Layer 2–3 containing three changed genes, and the CA1 region containing four changed genes (Table 12). The distinct patterns of expression changes for each area were best visualized following hierarchical clustering (Fig 7). In general, the top most changed genes were decreased by ethanol exposure. Another observation from the TREx results indicated that most of the significant expression changes found in the whole somatosensory cortex by RNA-Seq were not changed in the layer-specific TREx analysis, with a few notable exceptions. Specifically, Ei24, Errfi1, and Smad1 were changed in the whole somatosensory cortex as well in specific microdissected layers (denoted by asterisks in Table 12). This demonstrated that genes changes measured in whole brain region analyses may not be attributable to the whole region but rather to specific cell populations. It is also possible that inclusion of layer I and underlying white matter in the whole somatosensory cortex sample led to different results.

**Table 12:** TREx expression changes due to ethanol in WT mice for different brain regions, grouped by function.

Post-hoc contrast (p < 0.05): WT ^ethanol^ vs WT ^saline^	SOMATOSENSORY CORTEX				HIPPOCAMPUS	
GENE ONTOLOGY CATEGORY	Layer 2–3	Layer 4	Layer 5	Layer 6	CA1	DG
KEGG 04115: p53 signaling pathway	Ei24*, Rprm, Casp8^Ø^	Sesn1		Atm	Bai1, Cdk1, Pten, Tsc2	
GO:0006915 apoptosis	Ei24*, Casp8^Ø^, **Cdkn1b**, **Cd27**^Ø^, Jun, Nr2e1, **Parp1**	Sesn1, Egr1, Foxo3^Ø^, Cradd, **Sox9**, Xrcc4	Bcl2, Ctgf, Ddb1, Foxp1	Atm, **Cd27**^Ø^, **Cdkn1b**, Smad1*, Trp53bp2	Bcl2l1, Birc5, Casp6, Casp2, Pak7, Pten, **Sox9**	Dlx1, **Parp1**
GO:0007049 cell cycle	**Cdkn1b**, Jun, Nr2e1, Rprm	Hes1, Lig3, **Sox9**	Bcl2, Ddb1, Ctgf, **Smc1a**	Atm, **Cdkn1b**, **Smc1a**, Trp53bp2	Bcl2l1, Birc5, Pten, Casp2, Cdk1, Ppp3ca, Mapk1, **Sox9**	
GO:0006974 response to DNA damage stimulus	Parp1, Pcbp4		Ddb1	Atm		
GO:0006281 DNA repair	**Parp1**	Lig3, Xrcc4	Ddb1, **Smc1a**, **Tdg**	Atm, **Tdg**, **Smc1a**	Apex2, Cd27, Xpa	**Parp1**
GO:0050767 regulation of neurogenesis	Mib1, Nr2e1	Foxo3^Ø^, Hes1, **Sox9**, Xrcc4	Bcl2		Pten, **Sox9**	Dlx1
GO:0030182 neuron differentiation	Jun, Mib1, Nr2e1	Foxo3^Ø^, Hes1	Bcl2, Foxp1	Smad1*, Smad4, Dlx2	Pten, Sox9	Dlx1
GO:0007268 synaptic transmission	Nr2e1, **Syp**	Egr1, Sox9		Sst, **Syp**	Gad1, Kras, Pten, **Syp**	
GO:0043005 neuron projection	Casp8^Ø^, **Cd27**^Ø^			**Cd27**^Ø^, Syp	Pten, Tsc2, Ywhae	
GO:0048169 regulation of long-term neuronal synaptic plasticity		Egr1		Syp		
GO:0045321 leukocyte activation	**Cd27**^Ø^, Jun	Egr1, Hes1, Xrcc4	Bcl2, **Foxp1**	Atm, **Cd27**^Ø^	**Foxp1**	
GO:0050793 regulation of developmental process	Casp8^Ø^, **Cd27**^Ø^, **Cdkn1b**, Errfi1*, Jun, Mib1, Nr2e1, **Parp1**	Egr1, Hes1, **Sox9**, Xrcc4	Bcl2, Ctgf, **Foxp1**	**Cd27**^Ø^, **Cdkn1b**, Dlx2, Smad1*	Bai1, Casp6, Pten, **Sox9**	Dlx1, **Parp1**
GO:0016477 cell migration	Jun, Nr2e1	Egr1, Hes1, **Sox9**	Bcl2, Ctgf, Foxp1	Sst	Pak7, Pten, **Sox9**	

* Gene transcript also found significantly changed in total somatosensory RNA seq analysis

^Ø^ Gene transcript was not detected in total somatosensory RNA seq analysis, possibly due to poor read counts or gene not present in gene annotation

Genes in bold are significantly changed in more than one region

**Fig 8:**
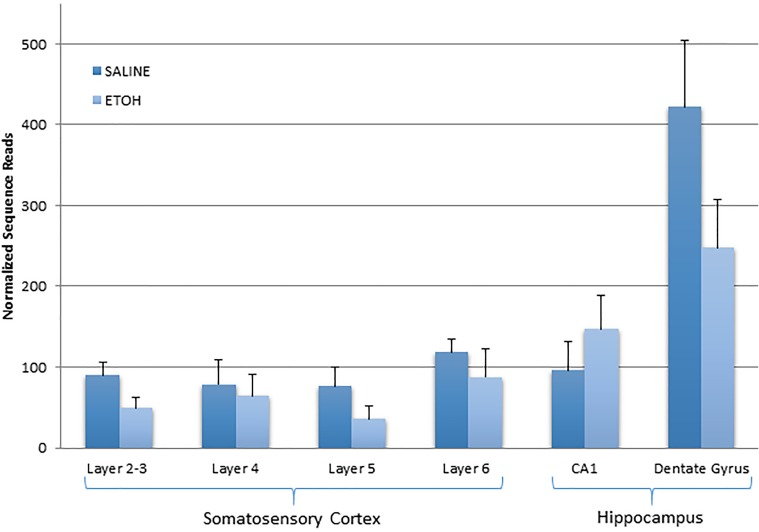
Expression of p53 transcript by targeted RNA-Seq in WT mice exposed to saline or ethanol. There is a general trend for decreased transcript expression in most brain regions following ethanol exposure (except for the CA1 region of the hippocampus). Bars show mean and T-bars indicate standard error of the mean n = 6 per group, four groups total.

**Fig 9:**
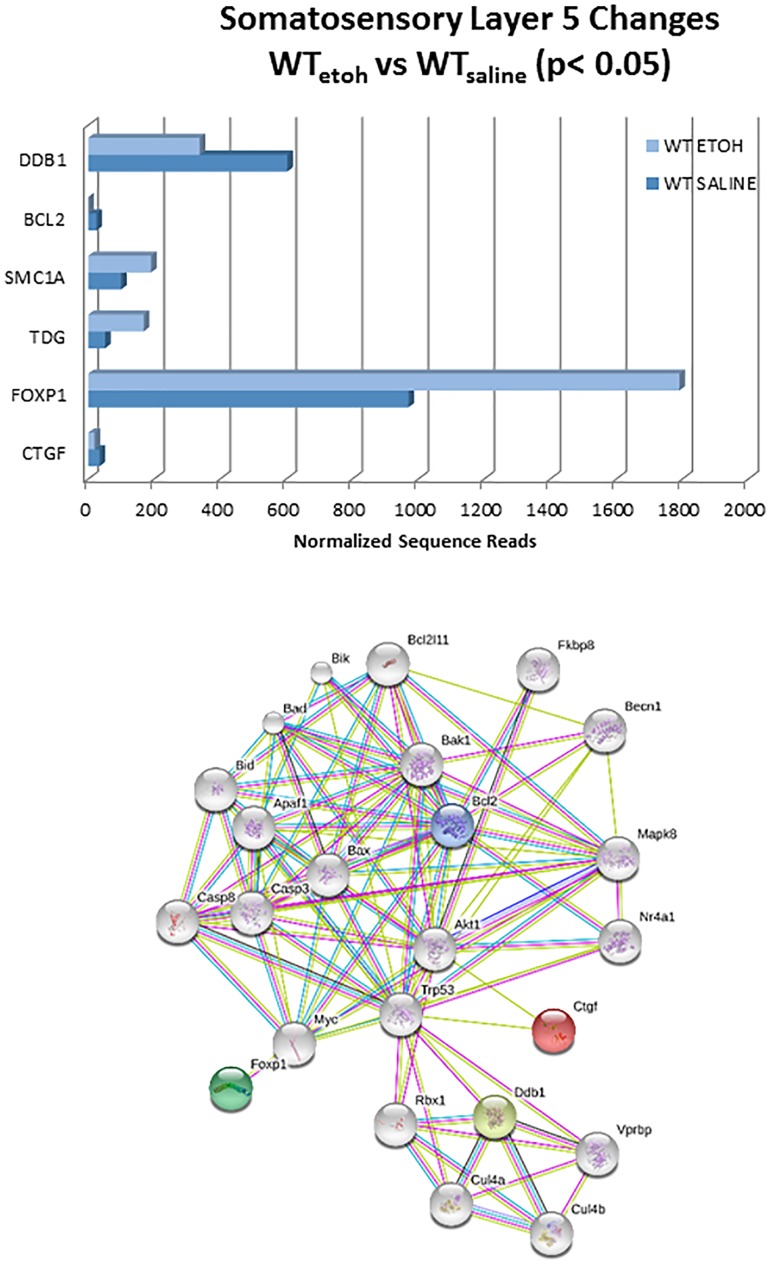
Expression changes in layer 5 of somatosensory cortex. Of the 280 genes assayed in the TREx, only 6 were changed in the WT mice exposed to ethanol. Of these, 4 were mapped in a single interaction network using the STRING database. Upper, normalized gene expression levels in TREx assay, with colors indicating the convention used for the KEGG TREx. Lower, STRING network for apoptosis. Note the presence of many genes that were assayed by TREx, but not found to be significantly changed (gray color). The genes that were changed are colored.

In addition to functional gene group analysis, we also examined the expression profile of the p53 transcript itself across multiple brain regions in the TREx data. These results indicated a somewhat consistent pattern of ethanol-induced decreases in five out of six of the regions (Fig 8). Moreover, the absolute basal levels of expression were higher in the dentate gyrus than the other brain regions examined. Based on the immunohistochemical images showing a possible increase in the numbers of apoptotic cells in layer 5 of the somatosensory cortex, we also specifically looked at the evidence for apoptosis-related gene expression changes in this specific area in the TREx data. Of the 280 genes assayed via TREx, only six were changed in layer 5 of the WT mice exposed to ethanol. However, of these, four were mapped to the KEGG apoptosis pathway (Table 12). The relationship of these genes to each other and to the other unchanged apoptosis-related genes was visualized using the STRING database interaction tool (Fig 9). This indicated an absence of direct gene-to-gene (or protein-to-protein) relationships within an otherwise densely interconnected network, that prominently featured p53 and caspase-3. The TREx data for the KO mice exposed to ethanol also showed that significant expression changes were present in each of the same functional categories, but the pattern differed across areas (Table 13). Several genes involved in multiple pathways tended to show up as changed in multiple regions in the KO mice. For example, Pten was changed in four of the six regions examined, and was also present in nine of the 13 functional categories. Similarly, Camk1 was changed in four of the six regions examined, and present in four of the 13 functional categories. And Nf1 was changed in three of the six regions examined and present in five of the 13 functional categories (Table 13).

**Table 13:** TREx expression changes due to ethanol in p53 KO mice for different brain regions, grouped by function.

Post-hoc contrast (p < 0.05): KO ^ethanol^ vs KO ^saline^	SOMATOSENSORY CORTEX				HIPPOCAMPUS	
GENE ONTOLOGY CATEGORY	Layer 2–3	Layer 4	Layer 5	Layer 6	CA1	DG
KEGG 04115: p53 signaling pathway	**Cdk1**, **Pten**	**Pten**	**Pten**	Ccne2	Bai1, **Cdk1**, **Pten**, Sesn2	Cdk4
GO:0006915 apoptosis	**Cdk4**, **Dlx1**, **Nf1**	**Jun**, **Polb**, **Pten**	**Pten**	Api5, Myc	Cdk1, Ctgf, Foxp1, **Jun**, Lig4, Mcl1, Nes, Nf1, **Polb**, **Pten**, Slk, Xpa	**Cdk4**, **Dlx1**, **Nf1**, Ppp1r13b
GO:0007049 cell cycle	**Camk1**, Ccnh, **Cdk4**, **Crebbp**, **Pms2**, Tada3	**Camk1**, **Jun**, **Pten**	**Pten**, Rpa3	**Camk1**, Ccne2^Ø^, Myc	Cdk1, Ctgf, **Jun**, Lig4, Mcl1, Nes, Ppp1r13b, **Pten**	**Camk1**, Ccnh, **Cdk4**, **Crebbp**, **Pms2**, Tada3
GO:0006974 response to DNA damage stimulus					Xpa	
GO:0006281 DNA repair	**Ccnh**, **Pms2**	**Polb**, Smug1	Rpa3		Actl6a, Cdk1, Lig4, **Polb**, Xpa	**Ccnh**, **Pms2**
GO:0050767 regulation of neurogenesis	**Camk1**, Jun, **Pten**	**Pten**, **Camk1**	**Pten**	**Camk1**, Myc	Lig4, Mib1, Nf1, **Pten**	**Camk1**, Dlx1, Nf1
GO:0030182 neuron differentiation	**Camk1**, **Dlx1**	**Camk1**, **Jun**, **Pten**	**Pten**	**Camk1**, Dcx*	Bai1, Foxp1, **Jun**, Lamc1, Mib1, Nes, **Pten**	**Camk1**, **Dlx1**
GO:0007268 synaptic transmission	**Nf1**, **Syp**	**Pten**, **Syp**	**Pten**		Gad1, **Nf1**, **Pten**	**Nf1**, **Syp**
GO:0043005 neuron projection	**Nf1**, **Syp**	**Pten**, **Syp**	**Pten**	Calb2, Myc	Gad1, **Nf1**, **Pten**	**Nf1**, **Syp**
GO:0048169 regulation of long-term neuronal synaptic plasticity	**Nf1**, **Syp**	**Syp**		Dcx*	**Nf1**	**Nf1**, **Syp**
GO:0045321 leukocyte activation		**Jun**			Foxp1, **Jun**, Lig4	
GO:0050793 regulation of developmental process	**Nf1**, Syp	**Camk1**, **Jun**, **Pten**	**Pten**	**Camk1**, Myc	Cdk1, Ctgf, Foxp1, **Jun**, Mib1, **Pten**	**Camk1**, Ccnh, Dlx1, Lig4, **Nf1**
GO:0016477 cell migration	**Nf1**	**Jun**, **Pten**	**Pten**	Dcx*, Myc	Cdk1, Ctgf, Foxp1, **Jun**, Lamc1, **Nf1**, **Pten**, Slk	**Nf1**

Conventions same as Table 12

To formally probe the evidence for ethanol-induced effects that were influenced by the genotype of the mice, we examined the TREx data for evidence of Treatment x Genotype interaction effects. The results identified a much smaller set of genes (Table 14). Of the genes with ethanol-induced expression changes in the presence of p53 protein, most were concentrated in the neuronal category and not the apoptosis category, except in the CA1 region of the hippocampus (Table 14).

**Table 14:** TREx expression changes due to interaction of ethanol treatment and genotype in WT and KO mice.

** **	SOMATOSENSORY CORTEX				HIPPOCAMPUS	
2 way ANOVA (p < 0.05): GENOTYPE x TREATMENT						
GENE ONTOLOGY CATEGORY	Layer 2–3	Layer 4	Layer 5	Layer 6	CA1	DG
KEGG 04115: p53 signaling pathway					Cdk1	
GO:0006915 apoptosis					Casp2, Cdk1, Stat1	
GO:0007049 cell cycle			Ctgf	**Camk1**	Casp2, Cdk1	**Camk1**
GO:0006974 response to DNA damage stimulus						
GO:0006281 DNA repair					Cdk1	
GO:0050767 regulation of neurogenesis				Camk1		Camk1
GO:0030182 neuron differentiation	Ephb1			Camk1		Camk1
GO:0007268 synaptic transmission						
GO:0043005 neuron projection	Ephb1				Stat1	
GO:0048169 regulation of long-term neuronal synaptic plasticity						
GO:0045321 leukocyte activation	Ephb1					
GO:0050793 regulation of developmental process	Ephb1		Ctgf	Camk1	Cdk1, Stat1	Camk1
GO:0016477 cell migration	Ephb1		Ctgf		Cdk1	

**Fig 10:**
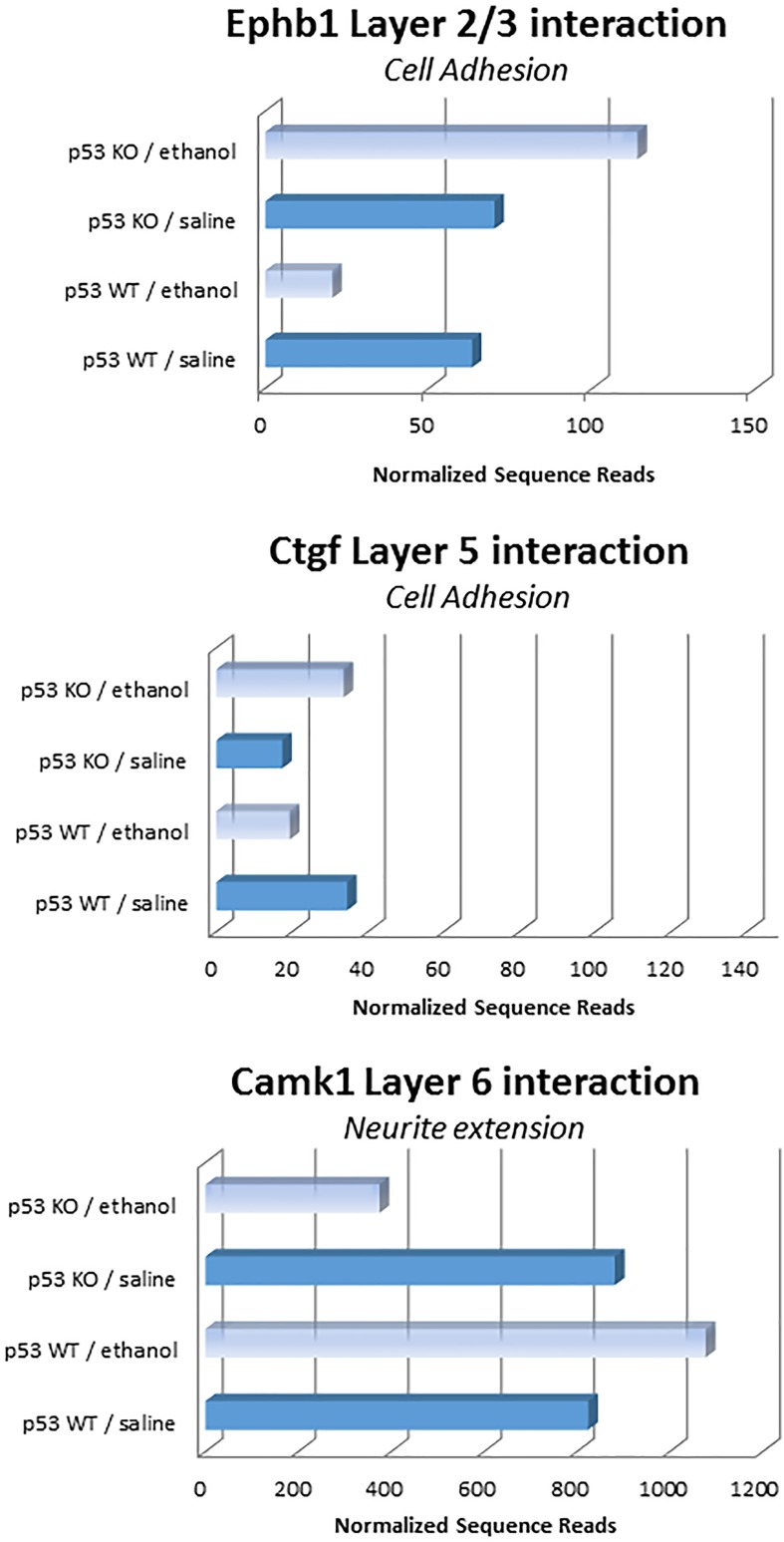
Genes with significant interactions between Ethanol Treatment and p53 Genotype Expression in subregions of the somatosensory cortex. Note that each of these 3 genes are involved in neuronal-related functions (cell adhesion or neurite extension. Note that for two of the genes (Ephb1, Ctgf) the direction of change in two different layers was an increase following ethanol in the p53 KO mice but a decrease following ethanol in the WT mice. The opposite pattern of change was seen in Layer 6 for Camk1 n = 6.

**Fig 11:**
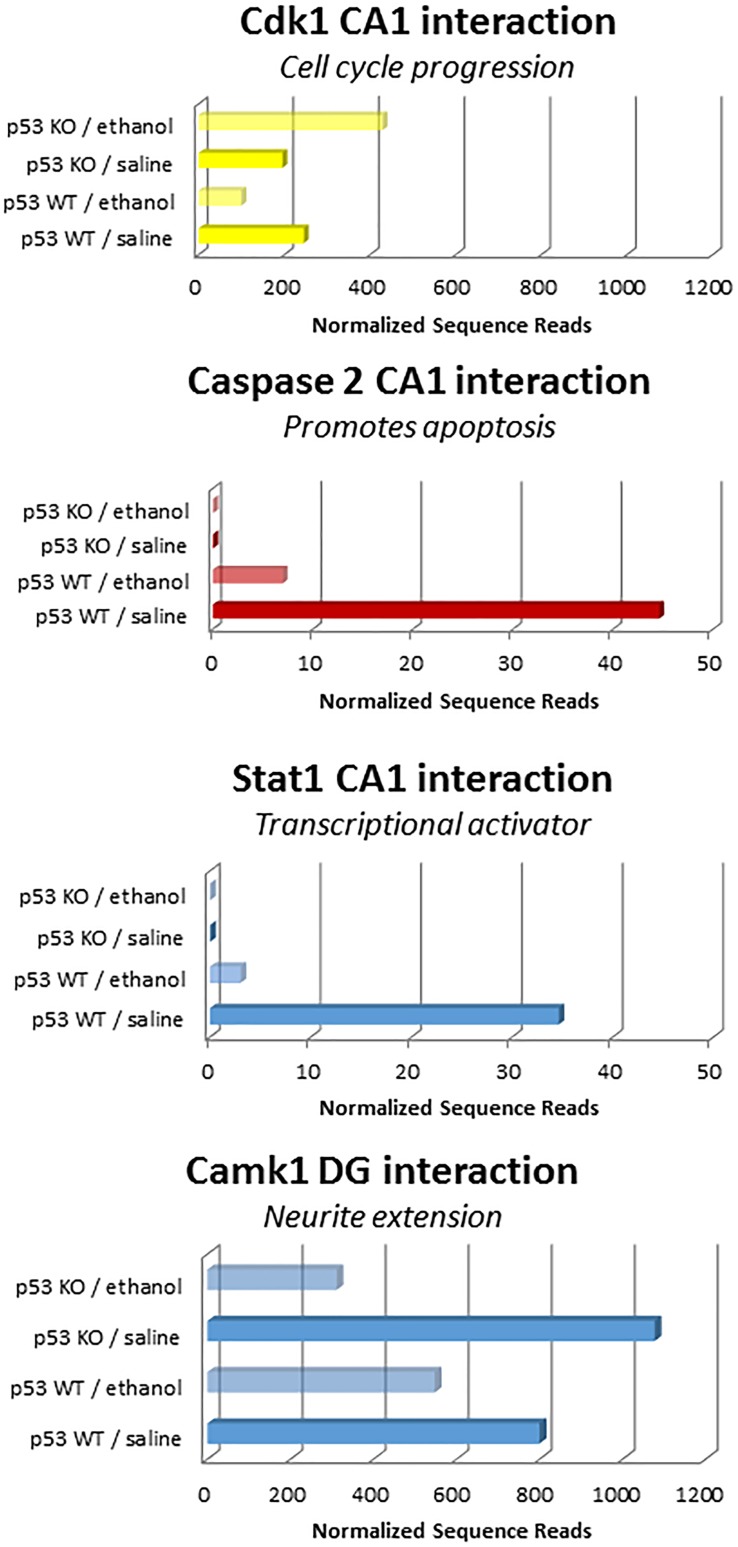
Genes with significant interactions between Ethanol Treatment and p53 Genotype Expression in subregions of the hippocampus. Two genes (Caspase 2 and State1) were not detected in the p53 KO mice, but showed decreased expression following ethanol administration in the WT mice CA1 regions. The Cdk1 gene showed opposite changes in response to ethanol in the p53 KO and WT mice (increased and decreased, respectively; top panel). The Camk1 gene showed a significant interaction of Treatment and Genotype that was due to a much larger decrease in expression in p53 KO mice after ethanol administration than in WT mice (bottom panel).

Surprisingly, layer 4 of the somatosensory cortex did not show any p53 interaction-related effects, although it did have many ethanol-related gene changes (Tables 12 and 13). Interestingly, Camk1 was the sole gene with interaction effects in layer 6 of the somatosensory cortex and the DG of the hippocampus, although it showed different directions of change in these brain regions (increased in layer 6, Fig 10; decreased in DG, Fig 11). In cortical layers 2–3 and layer 5, two genes involved in cell adhesion (Ephb1 and Ctgf, respectively) showed p53-dependent changes due to ethanol exposure (Fig 10). The CA1 subregion itself had the most robust interaction effects, with three distinct transcripts showing expression changes due to the interaction of ethanol and p53. These genes included Stat1, a transcription activator, caspase 2, a pro-apoptotic gene, and Cdk1, which promotes G1 and G2 progression. Two of these genes (caspase 2, Stat1) decreased in the presence of ethanol and p53 (Fig 11).

### P53 acetylation levels are unchanged by ethanol exposure

**Fig 12:**
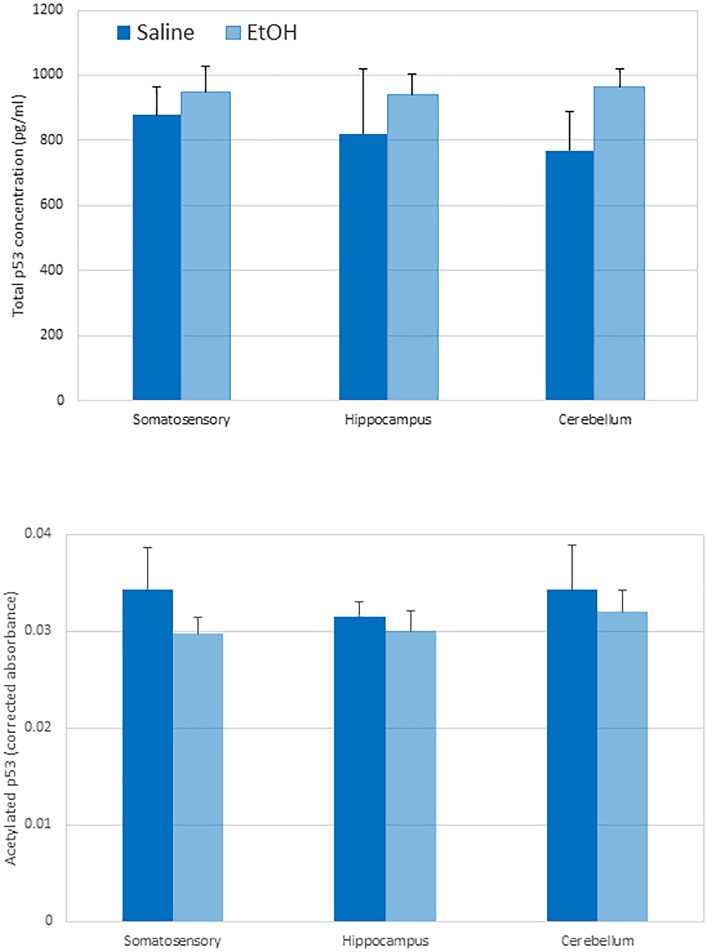
No change in the amount of total p53 or acetylated p53 levels due to ethanol exposure in WT mice. Upper, total p53 was detected using a pan-p53 antibody in an ELISA kit. Lower, acetylated p53 levels were detected using an acetylated p53 ELISA kit. Although total p53 increased and acetylated p53 decreased across the three brain regions in WT mice following ethanol exposure, the differences were not significant for any comparison. Mean and standard error of the mean are shown n = 3 per group.

Examination of total p53 and acetylated p53 levels indicated no significant changes due to genotype or ethanol exposure or their interaction (Fig 12), although the mean levels were lower in WT mice following ethanol exposure. Additional examination of the potential changes in the ratio of acetylated to total p53 also failed to demonstrate any significant differences.

## Discussion

Postnatal day 7 in mice is well established as part of a two week neuronal pruning and synaptic strengthening period in which some neurons are specifically destroyed in normal developmental process in the brain [29]. This period parallels parts of the second and third trimester period in human fetal development, which is why it is commonly used to model FASD, as we have done in the present study. Expression of the protein p53 is thought to be altered by ethanol, as well as other exogenous agents. These changes can promote either cell cycle arrest or apoptosis depending on the age and physiological state of the cell [21,30–32]. We hypothesized that part of the apoptotic response to ethanol was dependent on the presence of p53, or its downstream signaling. This hypothesis was examined using unbiased neuroanatomical immunohistochemical counting methods and transcriptome-wide molecular profiling.

We present four major findings. First, the vast majority of cells in the brain do not undergo apoptosis at the time point examined after ethanol exposure, and the presence or absence of p53 does not significantly alter the levels of apoptosis as measured in two different established methods. Nonetheless, the cell death observed is statistically greater than the basal levels that are occurring during this developmental period of synaptogenesis. Second, there are robust changes in genes related to cell cycling, DNA damage and repair, neuronal development, and synaptic function that occur in response to ethanol treatment, and some of these are strongly dependent on, or affected by, the presence of the p53 protein. Third, the specific genes and functional pathways that are altered by ethanol exposure and influenced by p53 are different in somatosensory cortex and hippocampus. And finally, there are no overall changes in these brain regions in the levels of total p53 or acetylated p53 following ethanol exposure in WT mice. In the sections that follow, we briefly review and discuss the implications of these findings.

### Apoptosis following ethanol exposure in P7 mouse model of FASD

We measured apoptosis using two different established methods–cleaved caspase 3 and ApopTag immunohistochemistry. Interestingly, we observed greater ApopTag staining than caspase 3 staining across all regions examined. This observation may be due to the fact that not all DNA damage (which is measured by ApopTag) leads to apoptosis or the fact that caspase 3 has yet to be activated in some cells or it may only be active for part of the cell death timeline, and the timing of the insult may change this. In fact, some of this damage may be repaired. In any case, the initial DNA damage is likely upstream of the active apoptotic protein cleavage activity in the cell [33]. Despite the overall differences, however, our data show that the results from both methods are well-correlated, and thus remain as reliable markers of apoptosis.

Our observations in the somatosensory cortex and the hippocampus also confirm findings from previous studies. Basal cell death assessed by ApopTag staining was approximately 0.4% in both genotypes, and this increased to approximately 1.45% following ethanol exposure, representing a 3.5 fold-increase. The average baseline cell death across brain regions assessed by active caspase 3 was 0.07% in both genotypes, and this increased to 0.76%, representing an 11-fold increase. Thus, ethanol clearly leads to increased apoptosis of a small subset of cells in the P7 brain, 8 hours after exposure.

Among the brain subregions examined, the dentate gyrus showed the smallest induction in apoptosis compared to baseline. Moreover, this was strongly influenced by the presence of p53, as it was the sole subregion showing an interaction effect between p53 genotype and ethanol exposure. One reason for the possible differences between the DG and other brain regions is the fact that this brain area retains populations of highly proliferative neural stem cells in the postnatal time periods.

### Gene expression changes related to cell cycling, neuronal activity and cell metabolism

To gain insight into the molecular changes induced by acute ethanol exposure, we performed transcriptome analysis of the whole somatosensory cortex using RNA-Seq. We found that many genes were significantly changed as a result of ethanol exposure, namely 14% of the 15,248 transcripts that were queried according to RefSeq annotation. A much smaller percentage of these changes were related to the presence of p53, with only 23 genes surviving multiple testing correction for the main effect of genotype at a cutoff of q<0.30. These data suggest that p53 is not the main focus of ethanol-induced genomic responses.

In an attempt to characterize the effect of p53 on genomic responses following ethanol exposure, the top significantly changed genes were examined in detail using a two-way ANOVA. Interestingly, the most robustly changed single genes were not related to apoptosis as some previous research has indicated in whole brain analyses [11, 34]. Rather, changes in gene expression due to ethanol exposure and the presence of p53 were grouped into functional categories related to the cell cycle, cellular metabolism, and neurodevelopment and neural activity. Possible explanations for these findings could be related to the known effect that ethanol has on cell cycle progression, neuronal activity (it acts on both GABA and NMDA receptors), as well as cellular metabolism and oxidative balance [35].

Categories related to the cell cycle that figured prominently in the top expression changes included Cell Cycle, Hippo Signaling Pathway, and several cancer-related pathways, including Glioma, Prostate Cancer, Endometrial Cancer, Colorectal Cancer, and Thyroid Cancer (Table 9). We point out that most of these cancer pathways reflect over-representation of cell cycle genes, and should not be interpreted as establishing a relationship between a particular end organ cancer pathway (as defined by KEGG) and the brain.

In terms of the effect on cellular metabolism genes, we note that the intracellular microenvironment is normally a reducing one, but alcohol dramatically changes this into an oxidative one [36–39]. Evidence for this can be inferred from expression changes that mapped onto categories including Butanoate Metabolism (KEGG ID 157, p = 0.00074) and also Synthesis and Degradation of Ketone Bodies (KEGG ID 63, p = 0.00157). Butanoate metabolism is centered on the production of butyrate, an important intermediate molecule with widespread cellular effects. For example, butyrate acts as a source of ATP during fatty acid metabolism, as a “nutrient sensor” through its binding to free fatty acid receptors, and as an inhibitor of histone deacetylases (HDACs), thereby promoting gene expression. Metabolic pathways such as the Synthesis and Degradation of Ketone Bodies could be related to multiple intracellular processes (including fatty acid metabolism, citric acid cycle, and glycolysis), but are also directly affected by the secondary and tertiary breakdown products of ethanol itself, including acetone. Thus, the effects on both of these pathways could reflect an altered energy homeostasis and redox state as a result of alcohol exposure.

We do not believe that the effects on the estrogen signaling pathways and related steroid biosynthesis networks (Tables 6, 7 and 9) are directly related to regulation of sex hormones *per se*. Rather, we believe that these changes likely reflect the involvement of estrogen signaling as a pro-survival or anti-apoptotic factor as well as a regulator of and cell cycle signaling. In fact, of the 24 genes involved in apoptosis that showed changes in the targeted RNA sequencing of individual brain subregions (Table 12), 9 are annotated as belonging to the Response to Lipid Gene Ontology (GO ID 0033993: Bcl2, Casp8, Cdk1, Ctgf, Jun, Kras, Nr2e1, Pten, and Sox9), 7 map to the Response to Steroid Hormone Gene Ontology (GO ID 0048545: Bcl2, Casp8, Cdk1, Ctgf, Kras, Nr2e1, and Pten) and 4 map to the Response to Estradiol Gene Ontology (GO ID: 0032355: Casp8, Cdk1, Ctgf and Pten). Moreover, Caspase 3 (Casp3) is also known to be responsive to estradiol and at least showed changes at the protein level in selected brain regions (Fig 2; Table 1). Thus, there is clearly cross-talk between cellular pathways affected by ethanol and p53 and those involved in lipid, steroid, estrogen and apoptotic signaling. Many of the changes noted such as increased expression of Steroid, Progesterone, and Estrogen genes in p53KO versus WT mice (Table 9) could represent potential compensatory mechanisms to mitigate the effects of loss of p53 by reducing apoptosis under baseline conditions (e.g., by modifying Bcl2 expression). Indeed, Bcl2 is widely recognized as a direct target of p53 itself.

Among the ethanol-induced expression changes related to neurodevelopment and neural activity that were also influenced by p53 genotype, we observed considerable enrichment of genes in several brain-specific pathways related to synaptic activity, including: Long-Term Potentiation, Dopaminergic Synapse, GABAergic Synapse, as well as Axon Guidance, and Neurotrophin Signaling Pathway (Table 9). Collectively, these observations suggest that p53 signaling impacts a wide range of processes that are considered essential for normal brain development and function and are also changed by ethanol.

### Heterogeneous gene expression changes in different brain regions in p53-related genes

To explore the p53 related molecular changes in more detail, we microdissected the individual layers of the somatosensory cortex and two of the subregions of the hippocampus. As in the whole somatosensory cortex profiling, the gene expression changes that were attributed to the presence of p53 comprised a relatively small fraction of the total ethanol-induced changes and included only six out of 280 queried genes. Surprisingly, we found that the brain-related genes that were identified with the presence of p53 tended to predominate. The predicted cell cycle changes were also identified in four out of six subregions of interest. Apoptosis-related gene expression changes only appeared to function in a p53-dependent manner in the CA1 of the hippocampus, though other non-p53 related apoptosis pathways may be in play. In fact, p53 appeared to play a major role in the ethanol response in the CA1, more than any other subregion investigated, according to our analysis. In contrast we did not find any attributable role of p53 in the ethanol induced gene changes in somatosensory cortex layer 4, which receives prominent input from the thalamus and other cortical layers. These localized gene expression profiles were not evident from whole somatosensory cortex analysis, suggesting that p53 is part of distinct active pathways in each subregion. Surprisingly all subregions, even in adjacent layers, showed distinct molecular alterations as manifested by the different combinations of gene expression changes. This is evidence of the heterogeneity of cellular responses to ethanol in the brain.

### Trend for decreased p53 transcript expression, but no change in protein expression

Comparable to previous results from our lab, we found a trend toward a decrease in expression of p53 transcript in ethanol-exposed mouse brain [10]. The trend held in all areas investigated except for the hippocampal CA1 area. Nonetheless the decreases were not statistically significant. However, sequencing data also showed that while the N-terminus of the p53 gene is not expressed in the KO mice, a portion of the 3’mRNA -terminus is expressed (Fig 6), and its levels appear to increase in these mice following ethanol exposure. If the partial C-terminus sequence is transcribed into a truncated protein, such a protein would likely be without effect, since upon expression it would be a sterically- hindered peptide incapable of tetramerization and binding to the DNA consensus sequence, which is required for p53 to become a functional protein, thus maintaining p53 null activity (Jackson Laboratories (*Trp53^tm1Tyj^*). On the other hand, if the truncated 3’ mRNA is increased in expression, it could also serve as a “decoy” for microRNAs that would normally target this region of the p53 transcript (such as miR-122-5p). The net effect of this would be to reduce the ability of such miRNAs to interact with other targets. Indeed, changes in p53-related miRNAs have been consistently observed following ethanol exposure in human subjects as well as ethanol drinking rats and neural stem cell culture [40].

At the protein level, in the whole somatosensory cortex, hippocampus, and cerebellum, we found no change in pan p53 protein levels following ethanol exposure in WT mice. However, the absence of a significant change in total p53 by itself does not mean p53 was not activated upon exposure to ethanol [31–32, 41]. Constitutively activated p53 (not bound to its inhibitor MDM2) can be lethal in development [42], and we did not assess levels of MDM2 protein expression. Moreover, there are many possible post-secondary modifications that p53 may undergo [32]. Phosphorylation of ser15 in mice is how p53 becomes increasingly active and functions as a transcriptional modifier even in unperturbed cells [17]. Another change that has been reported in studies of ethanol exposure in mouse liver is increased levels of acetylated p53 [22, 24]. However, we did not find evidence of increases in acetylation in any of the three brain regions considered (somatosensory cortex, hippocampus, and cerebellum).

### Limitations

There are a number of limitations of the present study that should be pointed out. First, in the initial whole-transcriptome profiling of the whole somatosensory cortex, we were not able to correct for false discovery in one of the specific contrasts of interest (comparisons of ethanol-treated p53 KO mice with saline-treated KO mice). Thus, the genes identified in that screen for whole somatosensory cortex changes must be considered tentative. Second, we did not pursue validation of individual genes identified in the whole somatosensory cortex analysis. Rather, we focused on a more in-depth approach of examining the potential for layer specific changes in expression of genes in the somatosensory cortex as well as subregions of the hippocampus. Part of the rationale for this was based on the initial immunohistochemical results that appeared to indicate possible differences in caspase-3 immunoreactivity in different cortical layers. In these follow-up studies, the decision of which genes to pursue in our was based largely on consideration of the Pathway Enrichment analysis of single genes and on the GO ANOVA results of entire gene sets, which suggested that there was potential disruption of synaptic, immune, p53 and apoptosis related functions. In retrospect, more of the top individual genes from the whole somatosensory cortex could have been included along with whole somatosensory cortex RNA samples. Finally, the results reported herein are based only on male mice, and we do not know how the effects might differ in females.

## Conclusions

In general, a more profuse cell death response to a binge ethanol exposure was expected based on previous literature [43]. Nonetheless, we did observe a significant increase in apoptosis upon ethanol exposure. Moreover, we consistently found that the absence of p53 in C57BL6 mice did not protect against apoptosis in the brain in the specific areas we examined that have been shown to be particularly vulnerable to ethanol exposure. These results are supported by findings in Ghosh and colleagues [28] and suggests that pro-apoptotic signaling is occurring via a p53-independent pathway.

Our results indicate that p53 is likely to be involved in cell cycle-related gene expression changes in layers 5 and 6 of the somatosensory cortex as well as both the CA1 and DG of the hippocampus, the latter of which is a site of continual neurogenesis in postnatal mice. There is evidence from previous studies that p53 can play a role in differentiation and cell division in neuronal stem cell cultures [44]. In the somatosensory cortex, the p53 cell cycle signaling changes we observed may be occurring in neurons but we cannot discount that it may also be occurring in non-neuronal cell populations such as microglia and other cell types. Nonetheless, the fact that ethanol causes significant changes in cell cycle genes means it impacts both neurogenesis and gliogenesis, leading to devastating ramifications on brain circuitry formation and function.

The present study has identified that p53 is engaged in neurodevelopmental signaling pathways in all subregions interrogated except layer 4 of the somatosensory cortex. Changing neuronal projections in the early postnatal brain can have severe consequences as this is a particular time when synaptic connections are refined, which sets the foundation for functional brain activity throughout life [45]. Previous alcohol research has shown that there are physical changes in neuronal processes and neuronal spine densities [46] (reviewed in [4]). Moreover, decreases in spine density following ethanol exposure correlate with decreased spatial memory (reviewed in [4]). Thus, the observed p53 dependent changes in synaptic transcripts and cell cycle transcripts in the brain may collectively impair normal brain maturation and lead to the intellectual disability that is well established as a possible consequence of FASD.

Our results also highlight a relatively new category of p53 dependent signaling responses following ethanol exposure that relate to effects on fatty acid (butanoate in particular) and ketone body metabolism. This was made evident by expression changes in Butanoate Metabolism and Synthesis and Degradation of Ketone Bodies pathways. Although we did not examine the biochemical mechanisms in this study, alcohol can contribute to a multitude of oxidation reactions, and to fatty acid synthesis in the brain [47]. Moreover, p53 has also been implicated in fatty acid transport [48–49] (reviewed in [30]). Previous research has shown that changes in proteins that transport ketone bodies (produced from fatty acids) are associated with epilepsy and synaptic changes [50]. Coincidently, people with FASD have a high-comorbidity of epilepsy [51] (reviewed in [4]). In fact, efforts to control seizures using a ketone rich diet show some clinical benefit, and blood beta-hydroxybutyrate levels (an example of an oxidized ketone body) correlate with the degree of seizure control [52]. In addition fatty acid accumulation in a newborn’s meconium is a marker of ethanol exposure in second or third trimester of development [53].

Considering these findings, we propose a new model of the role of p53 following ethanol exposure in the developing brain. This model posits that there are developmental changes in p53 signaling which in turn cause a detrimental change in synaptic function or structure, and lead to long term deficits in intellectual and behavioral function. Furthermore, the mechanism for this effect may be related to a change in fatty acid and ketone body metabolism, although further research will be needed to test this possibility.

Finally, we wish to comment on the potential relevance of our findings using a third-trimester equivalent binge drinking model for human exposures. Few studies are available to provide precise statistics regarding the 2nd and 3rd trimester alcohol consumption patterns in women, and self-reported rates differ considerably in different countries. Nonetheless, there are strong indications that such drinking patterns pose a significant public health risk. For example, a recent study of 1577 Australian women found that almost half of the women who drank prior to pregnancy (46%) continued their weekly or binge drinking patterns into pregnancy, while 40% reduced their consumption and 14% stopped drinking completely [54]. Moreover, among the women who only binge drank prior to pregnancy, 55% were more likely to continue this pattern rather than reduce drinking (29%), and among the combined weekly and binge drinking group 61% continued to binge and 47% continued weekly drinking throughout their pregnancy. In the US, a study of 992 women in the California Pregnancy Cohort by Feldman and colleagues [55] reported that 89% of women drank alcohol during their 1st trimester, 40% drank during their 2nd trimester and 31% drank during their 3rd trimester. Furthermore, 25% of these women reported at least 1 binge drinking episode during their first trimester, which dropped to 8.5% during their 2nd trimester and 4% during their 3rd trimester. These numbers are consistent with more recent studies in the US involving much larger surveys of more than 311,000 women in the 2002–2009 Pregnancy Risk Assessment Monitoring System (PRAMS) dataset whose drinking patterns prior to pregnancy and during the last trimester of pregnancy were compared [56]. In that study, almost half of the surveyed women (49.4%) consumed alcohol before pregnancy, and among these women, 6.4% reported no reduction in drinking into their 3rd trimester, 6.6% reported some reduction of their drinking, and the remaining 87% reported that they stopped drinking altogether as of their 3rd trimester. Thus, even in this large US sample, 13% of women continued to drink through their 3rd trimester, although the percentage who binge drank is likely much smaller (in the 2–4% range based on the Feldman study). Consequently, the findings from our study, which was based on a single binge day exposure, holds potential relevance for at least 2–13% of pregnancies in the US alone.

## Methods

### Ethanol administration

All procedures involving animals were approved by the appropriate Institutional Animal Care and Use Committee (IACUC) at the University of Maryland, Baltimore, and were in accordance with NIH guidelines. p53 heterozygous knockout mice originally developed by Taconic and maintained on a C57Bl/6 background were obtained from Jackson Laboratories (*Trp53^tm1Tyj^*, strain 002101;Bar Harbor, ME). Permission to breed the mice was provided by Taconic. Mice were maintained on a 12 hour light/dark cycle (7 am on, 7 pm off). The wildtype (WT) and p53 knockout (p53 KO) offspring of the mating of male and female p53 heterozygotes were injected intraperitoneally with ethanol (EtOH, 2.5 g/kg, 20% v/v solution) on postnatal day 7 (P7; the day of birth was designated P0) between 8–10 am with a second injection administered 2 hr later. Prior work has established that this protocol produces blood ethanol concentrations exceeding 400 mg/dl within 30 min after the second injection. The control mice of both genotypes received equivalent volume injections of normal saline. Genotypes were confirmed by PCR using three primers:

ACAGCGTGGTGGTACCTTAT (present in both WT and p53 KO)
TATACTCAGAGCCGGCCT (WT-specific)
CTATCAGGACATAGCGTTGG (p53 KO-specific)

A total of 44 male mouse pups were injected in this study, divided equally (n = 11/group) into four treatment groups: (1) WT + Ethanol; (2) WT + Saline; (3) p53 KO + Ethanol; (4) p53 KO + Saline. Five mice in each group were used for visualization of ApopTag and active caspase 3 immunoreactivity, with the six remaining mice used for molecular assays.

### Immunohistochemistry and cell counting

Eight hr after the second injection, mice were euthanized by isoflurane anesthesia and decapitation (for fresh frozen specimens) or isoflurane anesthesia and transcardial perfusion with PBS followed by 4% paraformaldehyde in PBS and decapitation. Brains were removed and fixed in 4% paraformaldehyde overnight, then submerged in graded sucrose solutions (15%-30%) and frozen at -80°C. Brains were then cryosectioned at 12μM thickness in the coronal plane and sections stored at -80°C. For immunostaining, sections were thawed and air-dried, then submerged in PBS (5 min), 3% H_2_O_2_ in 80% methanol (10 min), PBS (2 x 5 min), and blocked in 1% Bovine Serum Albumin (BSA) with 5% Non-fat Dry Milk (NFDM) in 0.5% Triton in PBS (60 min). Slides were processed for either ApopTag™ or active caspase 3 immunoreactivity. ApopTag was visualized according to the manufacturer's protocol, in the *In Situ* Apoptosis Detection Kit S7100 (Millipore, Billerica, MA) with anti-digoxigenin conjugate for colorimetric staining. Active caspase 3 reactivity was detected by incubating the tissue with primary antibody (AF835, 1:100; R&D Minneapolis, MN) at 37°C for 2 hrs, rinsed in 0.5% Triton (3 x 5 min), then incubated with a biotinylated secondary antibody (anti-rabbit [1:250]; Santa Cruz Biotechnology, Dallas TX SC2004) for 60 min. Slides were washed in 0.5% Triton (2 x 10 min), and PBS (2 x 5 min). ABC reagent was applied according to the manufacturer’s instructions using the Vector Laboratories (Burlingame, CA) kit PK-7100, followed by a PBS wash (3 x 2 min). Peroxidase substrate solution was then applied containing 0.05% DAB Acros Organics (Thermo Fisher Scientific, Waltham MA) 868272-85-9, and 0.015% H_2_O_2_ (Sigma, St Louis, MO H1009) in PBS, pH 7.2, until adequate color development (at least 30 sec). Slides were then rinsed in PBS (3 x 1 min) and counterstained with methyl green. All slides were then rinsed in three changes of distilled water, rapidly dehydrated in 95% ethanol, 100% ethanol and then cleared in xylene. Coverslips were then mounted on the glass slides with resinous mounting medium Cytoseal 280 8311–4 (Thermo Scientific, Waltham, MA). For quantification, the sections were examined using an Olympus (Center Valley, PA) BX53 light microscope at 20x for counting, and images were taken at 10x and 20x. Counts were done blinded to genotype and treatment using BioQuant (Nashville, TN) software (2013 version, BIOQUANT Image Analysis Corporation, Nashville TN) by random selection of 100 μM^2^ areas in the primary somatosensory cortex and three subregions of the hippocampal formation, CA1, CA3 and the dentate gyrus.

Initial statistical analysis of the cell count data was performed using a two-way (Genotype x Treatment) analysis of variance (ANOVA). Because of the low cell counts of immunopositive cells in several of the treatment groups, and the tendency for non-normal distribution of these data, the post-hoc contrasts were examined using a non-parametric Mann-Whitney test, with significance set at p < 0.05 for between-group comparisons. We also examined the correlation in the count data between the active caspase 3 and ApopTag methods using the Spearman correlation.

### Whole-transcriptome profiling of somatosensory cortex

We first used stranded RNA-Sequencing (RNA-Seq) to examine global gene expression changes in the four treatment groups (n = 6/group). Brains used for these studies were extracted and rapidly frozen at -80°C at 8 hrs after the second ethanol injection. Brains were shipped to SUNY Upstate Medical University for processing. Initially, we dissected the primary somatosensory cortex after crysectioning brains in the sagittal plane at 20 μM thickness and rapidly mounting them on PENfoil slides (Leica, Wetzlar, Germany) before performing a cytoarchitectural stain using the HistoGene (ThermoFisher Scientific, Waltham, MA) stain and procedure (Arcturus). Stained sections were compared with the Allen Developing Mouse Brain Atlas cytoarchitectural images and the somatosensory cortex dissected using the AS LMD laser dissection microscope (Leica) into PCR tubes containing Qiazol solution (Qiagen, Valencia, CA). These tubes were stored frozen at -20°C until purification according to manufacturer protocol using a two step procedure that combined the Trizol method for DNA, RNA, and protein purification followed by the RNeasy method for clean-up of the RNA. After performing the laser microdissection on half of the samples, the subsequent collections of somatosensory cortex were obtained using a hole-punch method on the same cortical areas in frozen brain specimens. These were also collected into Qiazol and the RNA purified in the same manner as before. All purified RNA samples were subsequently assessed to determine the yield, purity, and quality of the RNA using a Nanodrop (ThermoFisher) and the Agilent Technologies (Santa Clara, CA) RNA 6000 Pico Kit.

For whole-transcriptome analysis, three of the RNA samples that passed quality control standards (RNA Integrity Number ≥ 7) in each treatment group (n = 12 total) were submitted to the SUNY Molecular Analysis Core for Stranded Total RNA-Seq. Libraries were prepared using standard Illumina methods and sequenced with an Illumina (San Diego, CA) NextSeq 500 instrument using 1 x 75 bp single end reads at a targeted depth ≥ 15 million (M) reads per sample. Raw FASTQ files from the sequencing were generated in Illumina BaseSpace for initial quality control (filtering out reads with a q score < 30) and alignment was performed to version mm10 of the mouse genome and annotated to RefSeq (5-2-16) using the Bowtie2 algorithm within the TopHat application.

The aligned BAM files were downloaded into Partek Flow and Genomics Suite (St Louis, MO) for downstream analysis. The data summarization, quantification, and normalization of RNA counts into total reads per kilobase of gene model per million reads (RPKM) and initial single statistical analysis were performed in Partek Flow. Raw FASTQ files and normalized RPKM data are freely available in the NCBI Gene Expression Omnibus (Accession GSE100217). To identify single genes and transcripts with changes in expression, we used a two-way ANOVA to test for significant main effects of the two factors ethanol Treatment and p53 Genotype, as well as the interaction of these two factors. Whenever possible, false discovery rate (FDR) adjustment for multiple testing was performed on the main and interaction effects, with a cutoff of 0.3 or less initially used for single gene discovery in the comparisons of ethanol effects in WT mice, as well as the genotype effects and the ethanol x genotype interaction effects. Notably, no FDR cutoff was used for the ethanol contrasts in the KO mice due to less robust effects. For these comparisons, the top 25 genes were shown based on uncorrected nominal significance. False discovery rate (FDR) adjustment for multiple testing was performed on the main and interaction effects, with a cutoff of 0.3 or less used for single gene findings. Post-hoc contrasts were obtained using a Fisher's Protected Least Significant Difference, without correction for multiple testing. Functional enrichment analysis of the 50 most-robustly affected genes for each contrast of interest was performed based on the Gene Ontology (GO) and KEGG (Kyoto Encyclopedia of Genes and Genomes) annotation using Partek Pathway. We also transferred the normalized data from Partek Flow into Partek Genomics Suite to perform Pathway ANOVA using the KEGG annotation database. This procedure tests for shifts in the expression of all of the transcripts represented in a particular gene set, rather than annotating the most robustly-changed in a particular contrast.

### Targeted RNA-seq analysis of expression changes in selected cell populations

Laser dissected RNA samples of discrete lamina in the somatosensory cortex (layers 2–3 combined, layer 4, layer 5, layer 6) and discrete regions of the hippocampus (CA1 and dentate gyrus) were obtained using the previously described staining methods, with the exception that the dissected cells were collected into RLT Lysis Buffer from the RNeasy Kit (Qiagen, Valencia, CA). These RNA samples (6 per group for each region of interest, n = 144 samples total) were then subjected to Targeted RNA-Seq Expression (TREx) analysis according to the manufacturer’s protocol.

The content of the TREx panel included 280 genes involved in neuronal activity, apoptosis, DNA damage and repair, cell cycle regulation, and cell fate, plus housekeeping genes (S1 Table). These libraries were sequenced using the Illumina MiSeq instrument, with reads mapped to the mouse genome, normalized to the mean counts of peptidylprolyl isomerase A (PPIA) and hypoxanthine phosphoribosyltransferase (HPRT), and subsequently analyzed using a two-way ANOVA and t-test procedures for each brain region of interest in Metaboanalyst software. The 25 most robustly affected genes in each brain region comparison were subjected to hierarchical clustering for display purposes.

### Protein assays

For protein analysis, fresh frozen brains were thawed to 20°C and the left hemisphere was dissected. Partial samples of somatosensory cortex, hippocampus, and the cerebellum were obtained using a beveled 16G needle as a hole punch. These specimens were immediately placed in 500 μL of Qiazol (Qiagen, Valencia, CA). Protein/DNA/RNA purification was done following the Trizol extraction method. Protein was reconstituted in 50 μL of 1% SDS with 1:100 protease inhibitor cocktail and quantified using the Micro BCA Protein Assay Kit (Pierce, Waltham, MA) and measured on a plate reader at 562nm. Total p53 was measured using 1 μg total protein with the MyBioSource ELISA kit San Diego, CA (Cat. # MBS076677). Acetylated p53 was measured using 1 μg total protein with the Cell Signaling Technology (Danvers, MA) PathScan Acetylated p53 sandwich ELISA kit (Cat. #7236). Analysis of the protein data was performed using a two-way ANOVA to test for significant main effects of ethanol Treatment and p53 Genotype, as well as their interaction.

## Supporting information

S1 Table 280 genes in the Targeted RNA Seq (TREx) assay. (XLSX)
